# Descriptive analysis of national bovine viral diarrhoea test data in England (2016–2023)

**DOI:** 10.1002/vetr.5325

**Published:** 2025-05-30

**Authors:** Naomi S. Prosser, Edward M. Hill, Lorna Gow, Massimo Cavallaro, Michael J. Tildesley, Matt J. Keeling, Jasmeet Kaler, Eamonn Ferguson, Martin J. Green

**Affiliations:** ^1^ School of Veterinary Medicine and Science University of Nottingham Sutton Bonington UK; ^2^ Institute of Population Health University of Liverpool Liverpool UK; ^3^ NIHR Health Protection Research Unit in Gastrointestinal Infections University of Liverpool Liverpool UK; ^4^ Zeeman Institute for Systems Biology and Infectious Disease Epidemiology University of Warwick Coventry UK; ^5^ BVDFree England Scheme Coventry UK; ^6^ Agriculture and Horticulture Development Board Coventry UK; ^7^ School of Life Sciences University of Warwick Coventry UK; ^8^ School of Psychology University of Nottingham Nottingham UK; ^9^ NIHR Blood and Transplant Research Unit in Donor Health and Behaviour University of Cambridge Cambridge UK

## Abstract

**Background:**

Bovine viral diarrhoea (BVD) is an endemic disease in the UK. In England, a voluntary control and eradication scheme, BVDFree England, has been running since 2016.

**Methods:**

We analysed test results from 7005 herds that were submitted to BVDFree England between 2016 and 2023 to investigate changes in the prevalence of BVD in participating herds and engagement by farmers since the previously published analysis covering the period up to 2020.

**Results:**

Herds that tested for multiple consecutive years were more likely to be BVD negative in later testing years than when starting. Few herds were still positive after 5 years of testing. Overall, the prevalence of BVD‐positive herds in the dataset declined between 2020 and 2023; however, fewer farmers joined the scheme for the first time each year since 2019 (214 in 2023 compared with 2614 in 2019).

**Limitations:**

This dataset represents the herds that submit tests to BVDFree England and is not representative of all cattle herds in England.

**Conclusion:**

Herds that tested for multiple consecutive years in the scheme were less likely to be BVD positive in later years of testing, and the prevalence of BVD in participating herds has continued to fall since 2020.

## INTRODUCTION

Bovine viral diarrhoea (BVD) is one of the most economically important endemic cattle diseases in the UK.^1^ The disease results in reduced health and productivity of dairy cows through lower growth rate, fertility and milk yield and increased incidence of abortion and comorbidity.^2^, ^3^ BVD is caused by a pestivirus and is propagated via persistently infected (PI) calves, which are created when their dam becomes infected within the first 120–125 days of gestation.^4^ PI cattle shed virus throughout their life, while cattle that are infected with the virus after birth are only transiently infected and have long‐lasting immunity following infection.^5^ As a consequence of these epidemiological characteristics, eradication schemes focus on the identification and removal of PI cattle.^6^ There are two common approaches to identify PI animals: (1) individually testing animals for the virus or antigen with retesting of positive animals to distinguish persistent from transient infection, or (2) testing a sample of youngstock from each management group for antibodies to BVD, with individual virus/antigen testing of individuals within any group that tests positive to identify the PI animal(s). These two approaches have been successfully used in BVD eradication schemes in various countries.^7^, ^8^

Within the UK, each country has their own BVD eradication scheme, with mandatory testing in Scotland and Northern Ireland and voluntary testing in England and Wales.^9^ England had a voluntary scheme (BVDFree England) that came to a close on 31 July 2024. In the BVDFree England scheme, farmers could submit BVD test results to obtain a herd status after 2 years of testing following the scheme requirements. England now has the Animal Health and Welfare Pathway scheme, which was initiated in 2023.^10^ The pathway funds an annual health and welfare review from a veterinarian and some BVD testing. This has been available from February 2023 for farmers with 11 or more cattle, with further financial support available for prevention and reduction of BVD on farms.^10^

A previous analysis of the test results submitted to BVDFree England was conducted on the test results up to and including 2020.^11^ To assess subsequent progress of the scheme and to assess the prevalence of BVD in herds that tested consecutively over multiple years, the aim of this research was to conduct further analysis of the data, including test results up to the end of 2023.

## METHODS

### Data source and cleaning

The BVDFree England dataset available included 1,409,517 test results from samples taken by 7006 herds between 2016 and 2023 (correct as of 21 February 2024). The herds submitting tests to BVDFree England were not representative of all English herds, and herds may have been testing for BVD for different reasons. We do not know the rationale farmers had for testing in the scheme. Further information on the dataset can be found in a previous analysis of test results for 2016–2020.^11^ All data preparation and analysis were conducted in R statistical software (version 4.3.1),^12^ and data preparation was conducted using the same approach as for the previous analyses.^11^ Before conducting any analysis, we removed some tests with incorrect or missing data. We removed 285 tests that had a sample date after the automatically generated test result upload date and five tests that were not assigned to a herd, resulting in a dataset with 1,409,227 tests from 7005 herds. Some tests had herd‐level data missing. Postcode was missing for 1600 (22.8%) herds, the type of cattle (beef breeder/beef fattener/dairy/calf rearer) was missing for 1758 (25.1%) herds and herd size was missing for 1750 (25.0%) herds.

### Descriptive analysis

Herds were split into the two testing regimes that must be adhered to in order to get a herd result from BVDFree England: virus testing (‘tag and test’) of individual calves and antibody testing of at least five animals in each youngstock management group.^13^ Herds were defined as ‘antibody testing’ if they only used individual antibody tests (a minimum of five tests, as required for youngstock cohort testing^13^) or as ‘virus testing’ if they only used individual antigen or virus tests and the number of tests conducted in a year was at least 60% of the number of breeding cows. For virus testing, we used a threshold of 60% of the number of cows because there was a bimodal distribution in the number of tests as a proportion of herd size, with the peak encompassing a proportion of 1 starting at 0.6 (Figure S1). For these herds, it was inferred that most calves were being tested using BVD antigen testing tags. We did not use a minimum number of tests in the previously published analysis of the dataset^11^; however, in this paper, references to virus testing herds uses the definition of herds testing at least 60% of the number of breeding cows on‐farm. A comparison of results obtained using a 60% of herd size minimum number of tests and no minimum number of tests are in Table S1. Note that some herds were not categorised into one of the two testing regimes based on the definitions we used. These herds were included in the overall descriptive statistics, but not when reporting the herds that were assigned to a testing regime. Herds were defined as BVD positive in a calendar year if, in that calendar year, they had at least one positive antigen, virus or antibody test result, and negative if, in that calendar year, none of the tests submitted had a positive test result.

We calculated summary statistics by test regime (virus or antibody), herd type (beef breeder only or dairy only) and calendar year. We visualised regional variation in the number of calendar years for which herds submitted tests to BVDFree England using heat maps and the trajectory of individual herd status over time using an alluvial plot.

### Models to assess BVD prevalence with continued engagement with the scheme

We fit two models to investigate the prevalence of BVD in herds with multiple years of testing in the BVDFree England scheme. In both models, we included only the herds that were testing for at least two consecutive years to investigate herds with continual engagement. Model 1 was a binomial mixed‐effects model to investigate the association between the odds of a herd being positive for BVD and the number of years testing (using either the virus or antibody testing route) in the scheme. Model 2 was a proportional binomial mixed‐effects model to investigate the association between the proportion of tests that were positive in a herd and the number of years a herd had tested for BVD using the virus testing route in the scheme. For model 2, we selected only virus testing herds as a proxy to estimate the within‐cohort prevalence of PI animals, which we were not able to calculate from antibody tests, as positive antibody test results could be due to past infection, maternal antibodies or, possibly, vaccination. We tested herd size (number of breeding cows), herd type and region of England as possible confounders in both models and assessed model fit using decile plots to compare the observed and predicted data.^14^

## RESULTS

### Participating herd summary statistics

**FIGURE 1:**
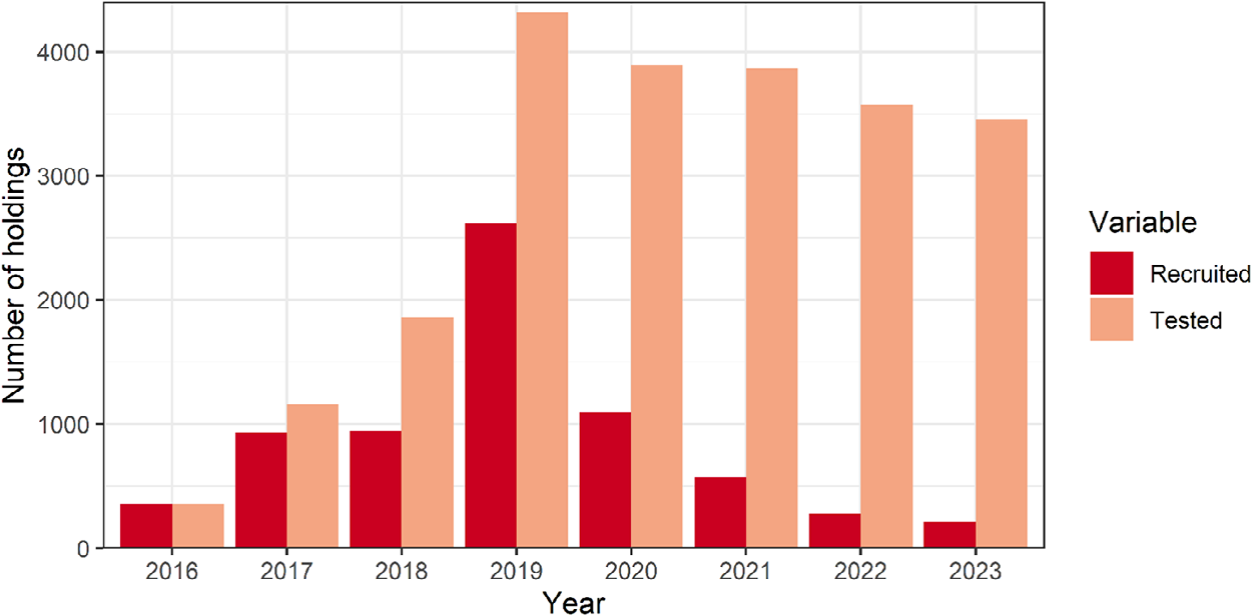
Number of holdings submitting tests to BVDFree England for the first time (recruited) and the total number of holdings submitting tests to BVDFree England each year (tested) from a total of 7005 herds that submitted tests between 2016 and 2023

A total of 7005 herds submitted tests to BVDFree England between 2016 and 2023, which is 20.9% of the 33,489 cattle holdings in England (in June 2023).^15^ Both the number of herds submitting tests for the first time and the total number of herds submitting tests have continued to decrease since the peak in 2019 (2614 herds recruited), with 571 herds recruited in 2021 and 214 herds recruited in 2023 (Figure 1). In 2023, a total of 3456 herds submitted tests to BVDFree England, which is 10.3% of cattle holdings.^15^ The median herd sizes in 2023 were 200 cows for dairy herds and 40 cows for beef breeding herds, which is similar to the 2020 median values of 195 dairy cows and 40 beef cows. Each year, there was a higher proportion of dairy herds than beef herds submitting tests to the scheme compared to the national proportion of 29% dairy and 71% beef.^15^ Specifically, in 2023, 60.0% of herds (of known herd type) that submitted tests to the BVDFree England scheme had dairy cows compared to 42.6% which had beef cows (5.2% had both dairy and beef cows and 2.6% had neither and were solely fattening animals and rearing calves).

**FIGURE 2:**
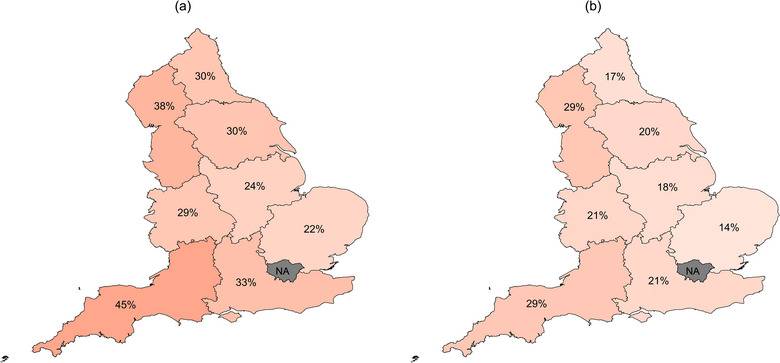
Proportion of breeding cows in each region of England that are within holdings that (a) have submitted a test to BVDFree England between 2016 and 2023 and (b) submitted a test to BVDFree England during 2023. The darkness of the colour directly correlates with the stated proportion for each region

The nine regions of England differed in the proportion of their breeding cows that were in herds that submitted at least one test to BVDFree England between 2016 and 2023. The lowest engagement across all years was in the East of England, where 22% of the breeding cows were in herds that had submitted test results to BVDFree England for at least 1 year, whereas the South West had the highest proportion at 45% (Figure 2a). In 2023, the regions with the greatest proportion of breeding cows in testing herds were the North West (29%) and South West (29%), and the region with the lowest proportion was the East of England (14%) (Figure 2b).

### National prevalence of BVD‐positive herds

There was little change in the proportion of herds using each testing regime since 2020. Dairy herds were more likely to use individual virus tests than beef herds. Overall, 39.4% of beef breeders were individual virus testing in 2023, compared with 32.1% in 2020, and 41.7% of dairy farmers were individual virus testing in 2023, compared with 44.0% in 2020. Since 2020, the proportion of the herds submitting test results to BVDFree England that had at least one positive test declined for both dairy and beef herds across both testing regimes (Figure S2). Within virus testing herds, there was little change in the proportion of tests within the herd that were positive between 2020 and 2023. The proportion of tests that were positive for BVD in all virus testing herds was 0.4% in 2020 and 0.3% in 2023, while the proportion of tests that were positive in BVD‐positive virus testing herds was 1.4% in 2020 and 1.5% in 2023 (Figure S3).

### Prevalence of BVD‐positive herds with consecutive years testing

**FIGURE 3:**
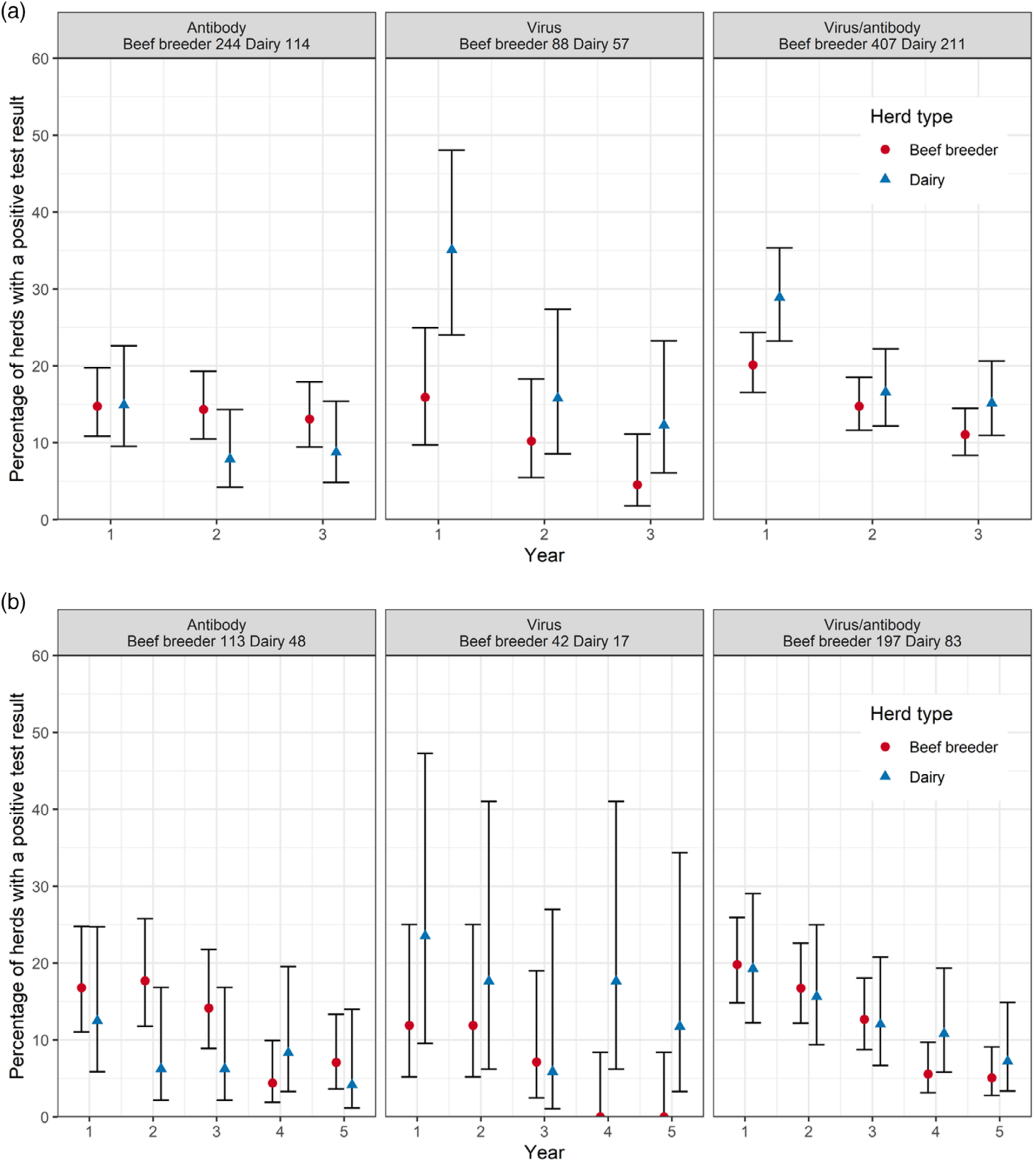
Percentage (point) and 95% confidence intervals (error bars) of herds submitting tests to BVDFree England every year for consecutive years that had at least one positive test result in year 1 for herds using antibody testing, virus testing or either testing regime for (a) 618 herds that submitted tests for three consecutive years and (b) 280 herds that submitted tests for five consecutive years. The numbers in the header beneath each testing regime denote the number of herds of that type for that testing regime

**FIGURE 4:**
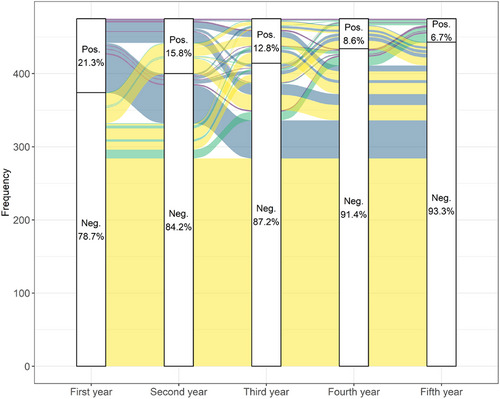
Alluvial plot illustrating changes in herd‐level bovine viral diarrhoea status (Pos.: positive, Neg.: negative) of 475 herds that used a testing regime (either virus or antibody) for five consecutive calendar years. Purple: positive at start and end of testing, blue: positive at start and negative at end of testing, green: negative at start and positive at end of testing and yellow: negative at start and end of testing

Few herds used the same testing regime for five consecutive years, so we present results for herds that tested for three consecutive years (Figure 3a) as well as for five consecutive years (Figure 3b). For herds that were testing consecutively for either 3 or 5 years, herds were more likely to have a negative BVD status as the number of years testing increased. For herds that underwent testing for three consecutive years, both virus and antibody testing herds were less likely to be positive in the third year of testing than the first year (Figure 3a). For herds that underwent testing for five consecutive years, 5.5% of beef breeder herds and 16.2% of dairy herds were positive in their fifth year of testing compared to 24.8% and 26.3%, respectively, in their first year of testing (Figure 3b). In model 1, the odds of a herd being BVD positive reduced for each consecutive year of testing for the first 5 years of testing, with herds having 0.19 times the odds of being BVD positive in the fifth year compared to the first year. For two to four consecutive years of testing, the odds of having BVD in each consecutive year of testing was significantly lower than for the preceding year of testing (odds ratio not within the 95% confidence interval of the odds ratio for the preceding year) (Table 1). Neither herd size, herd type or region were identified as confounders, and the model fit was deemed adequate (Figure S4). Figure 4 shows the change in herd status for each year of testing for the herds testing for five consecutive years. For herds that were positive for BVD in the first year of testing, 91.1% (92/101) had gone negative and 8.9% (9/101) remained positive or had become positive again by the fifth year. For the herds that were negative for BVD in the first year of testing, 93.9% (351/374) were negative and 6.1% (23/374) were positive by the fifth year.

**TABLE 1:** Results of model 1, a binomial mixed‐effects model to investigate the odds of a herd having at least one positive bovine viral diarrhoea test result, explained by the number of consecutive years testing (fixed effect) and herd (random effect), for 1799 herds that used a testing regime (either virus or antibody) for at least two consecutive years between 2016 and 2023

Number of consecutive years testing (reference = 1)	Number of herds	Odds ratio	95% confidence interval	*p*‐Value
2	1799	0.59	0.49–0.71	<0.001
3	1120	0.45	0.36–0.57	<0.001
4	772	0.28	0.20–0.38	<0.001
5	475	0.19	0.12–0.29	<0.001
6	218	0.23	0.13–0.40	<0.001
7	90	0.48	0.24–0.98	0.044
8	26	0.32	0.08–1.29	0.111

### Prevalence of BVD within herds with consecutive years testing

**FIGURE 5:**
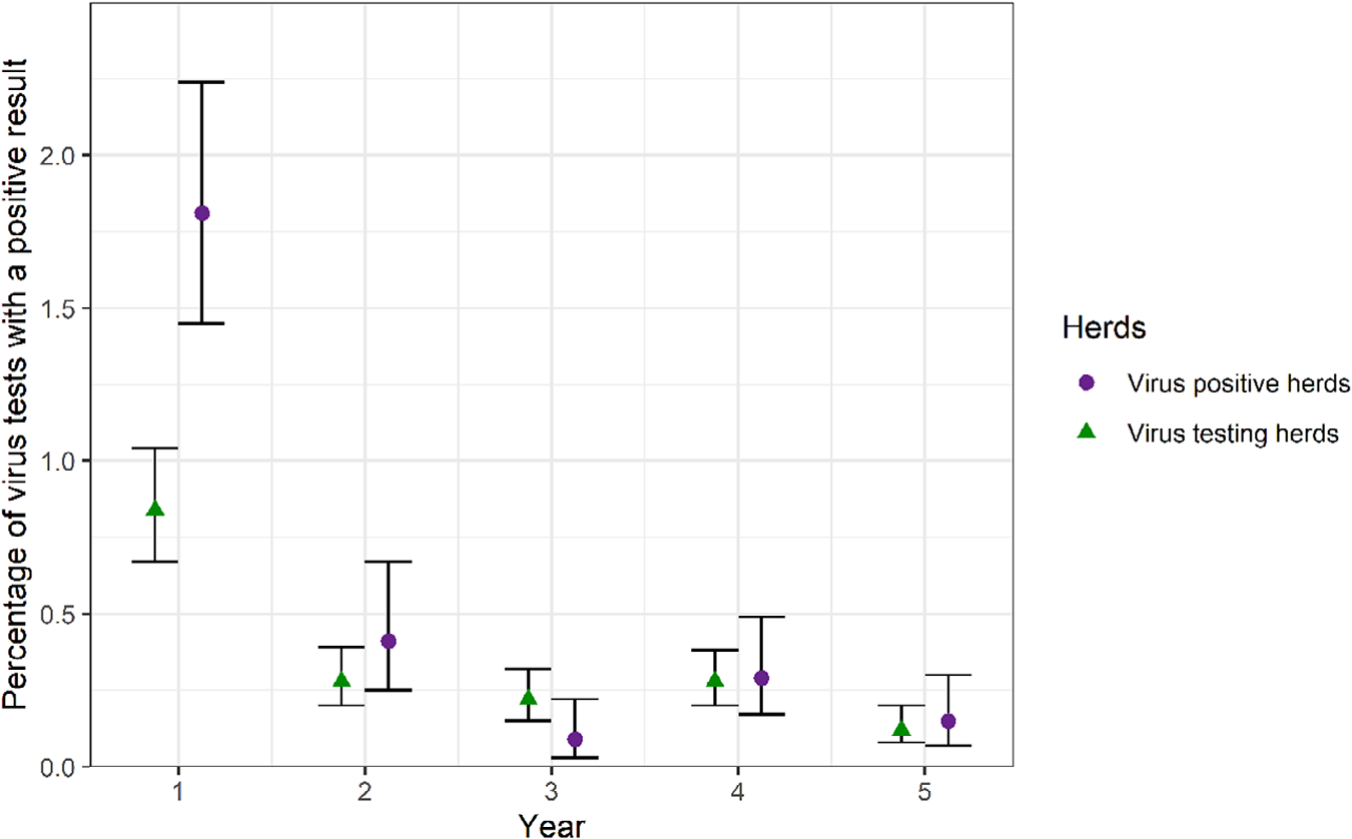
Percentage (point) and 95% confidence intervals (error bars) of tests that were positive from 141 virus testing herds submitting tests to BVDFree England every year for five consecutive years between 2016 and 2023. Virus positive herds consist of herds that were positive in their first year of testing, and virus testing herds consist of all virus testing herds that submitted tests for five consecutive years

The prevalence of BVD‐positive tests within testing herds fell with increasing number of years testing in the BVDFree England scheme; 0.1% of tests were positive in the fifth year of testing compared to 0.8% in the first year, and the decrease was steeper for herds that were positive in their first year, falling from 1.8% to 0.1% (Figure 5). In model 2, for the within‐herd prevalence of BVD‐positive test results, the proportion of positive tests tended to decrease as the number of years of testing increased, although the number of herds testing for more than 6 consecutive years was small and the confidence intervals of the related odds ratios were relatively large (Table 2). The odds ratio for the within‐herd prevalence of BVD generally overlapped with the 95% confidence interval of the next consecutive year of testing. Neither herd size, herd type or region were identified as confounders, and the model fit was deemed adequate (Figure S5).

**TABLE 2:** Results of a proportional binomial mixed‐effects model for the within‐herd prevalence of positive bovine viral diarrhoea test results, explained by the number of consecutive years testing (fixed effect) and herd (random effect), for 599 herds that were using the virus testing route for at least two consecutive years between 2016 and 2023

Number of consecutive years testing (reference = 1)	Number of herds	Odds ratio	95% confidence interval	*p*‐Value
2	599	0.47	0.39‒0.55	<0.001
3	373	0.42	0.33‒0.52	<0.001
4	261	0.44	0.34‒0.56	<0.001
5	141	0.18	0.11‒0.30	<0.001
6	73	0.15	0.07–0.31	<0.001
7	40	0.33	0.16‒0.67	0.002
8	13	0.21	0.06–0.70	0.011

## DISCUSSION

In this paper, we present an analysis of the test results submitted to BVDFree England up to 2023 to explore how BVD control has progressed in England since the last published analysis in 2020. We also investigated the effect of continued scheme involvement on the prevalence of BVD in herds that have committed to testing for a period of consecutive years.

Herds that consistently submitted tests to BVDFree England were more likely to be BVD free in later years of consecutive testing than in their first year of testing, with a large reduction in the prevalence of BVD in these herds after just a few years of testing. The testing regimes that BVD Free England use are based on regimes that have been used with success by other countries to achieve eradication or near‐eradication of BVD^16^, ^17^; therefore, it was not unexpected that consistent testing in a herd led to a lower prevalence of BVD compared to the first year of testing. A limitation of this study is that the herds that tested for multiple consecutive years were a relatively small subset of all herds submitting tests, and farmers may decide to stop or continue testing for BVD for a variety of reasons. There was a small proportion of herds that either remained positive throughout five consecutive years of testing, became briefly negative before becoming positive again, or became positive after initially being negative. It is not known why this has happened for each of these farms, but it could be due to incomplete identification and removal of all PI animals or reintroduction of BVD into a negative herd. A common barrier to herds achieving freedom from BVD within the UK and Ireland is farmer reluctance to remove PI animals.^17^, ^18^, ^19^ This can be due to reluctance to cull without compensation and instead attempting to raise the animal for slaughter or sale due to not understanding the production and financial implications of retaining PI animals.^20^, ^21^ Good biosecurity measures are also important to prevent the reintroduction of BVD into the herd.^22^, ^23^, ^24^ Legislation and financial incentives are key to encouraging all farmers to comply with PI animal removal, as well as ensuring that farmers understand the risk of retaining PI animals in their herd and engaging veterinarians and other stakeholders in BVD control, and these measures have successfully reduced PI animal retention by farmers in other countries.^17^, ^18^

We found that the prevalence of BVD‐positive herds in herds submitting tests to BVD Free England continued to decrease between 2020 and 2023, from 14.9% to 8.6% in beef breeder herds and from 21.9% to 14.5% for dairy herds. However, the herds submitting tests to BVDFree England were not representative of all English herds. Our analysis shows that these herds were typically larger and more likely to be dairy than English herds in general. Herds also may have been testing for BVD for different reasons. For instance, they may have been more likely to join either because they thought they had, or had not, got BVD in their herd. We also do not know from this dataset which farms were testing according to the requirements of BVDFree England and what the fate of PI cattle was. The decline in the proportion of herds infected with BVD suggests that most herds were removing PI cattle and not getting re‐infected. We do not know the rationale farmers had for testing in the scheme, but this would be valuable information for assessing a voluntary scheme. Understanding the rationale for farmers to test with BVDFree England and how they differ or not from the general population would help with interpretating how generalisable these results are, which is currently a limitation of this study.

There has been a decrease in the number of farmers joining the BVDFree England scheme each year since 2019, and herds in more cattle‐dense areas were more likely to join the scheme than those in less cattle‐dense areas. Farmers engage with BVDFree England through their veterinarian, and it may be that in areas with fewer cattle, the farmer's veterinarian may not be so engaged with national cattle schemes. Other research has found that the relationship with the veterinarian is important in how farmers prevent and control BVD.^25^ During the voluntary phase of the Irish BVD control scheme, there was a higher participation in the dairy‐producing areas than the beef‐producing areas.^21^ Currently, around 21% of English cattle herds have tested through BVDFree England at some point, which is similar to the farmer engagement with the Northern Irish and Irish schemes before they became mandatory.^17^, ^18^, ^19^ Wales achieved a much higher voluntary engagement rate up to 2022, with free youngstock antibody screening at the bovine tuberculosis test and some funding available for the detection of PI animals; however, identification and removal of the PI animals was very low.^9^, ^26^ Voluntary schemes rarely achieve high engagement rates from farmers and often progress to incentivising testing and compliance with PI animal removal through legislation, as Scotland, Northern Ireland and Ireland have all done.^9^ Keeping momentum in farmer engagement and progress in disease reduction is important to prevent farmers becoming disillusioned with the scheme.^18^ The new Animal Health and Welfare Pathway presents an opportunity to build on the progress of BVDFree England if it manages to create substantial additional farmer engagement in BVD testing and control. However, to date, there is no database for BVD testing within the Animal Health and Welfare Pathway, which will make assessment of progress in national BVD control difficult.

## CONCLUSION

In conclusion, there has been a continued reduction of the prevalence of BVD in herds in the BVDFree England test dataset between 2020 and 2023, and the subset of herds that tested consistently for multiple years were less likely to have a BVD‐positive test result in later years of testing than in their first year of testing. However, increased engagement with farmers via the recently established Animal Health and Welfare Pathway scheme will be required to make further progress in BVD control in England.

## AUTHOR CONTRIBUTIONS

Martin Green, Michael Tildesley, Jasmeet Kaler, Eamonn Ferguson and Matt Keeling were responsible for funding acquisition and supervision. Lorna Gow and Martin Green were involved in conceptualisation. Naomi Prosser and Martin Green were involved in the formal analysis, investigation and methodology. Naomi Prosser curated the data and wrote the original draft, and all authors contributed to project administration and the review and editing of the paper.

## CONFLICT OF INTEREST STATEMENT

Lorna Gow is employed by the Agriculture and Horticulture Development Board, and the dataset was provided by the Agriculture and Horticulture Development Board.

## ETHICS STATEMENT

Ethical approval was granted by the University of Warwick Biomedical and Scientific Research Ethics Committee (BSREC 100/19‐20) prior to commencement of the study.

## Supporting information



Supporting Information

Supporting Information

## Data Availability

Restrictions apply to the availability of these data, and interested parties should get in contact with the Agriculture and Horticulture Development Board. The data analysis code is available as Supporting Information supporting this paper.
