# Supplementary material for: Descriptive analysis of national bovine viral diarrhoea test data in England (2016–2023)
Source: Vet Rec. 2025 May 30;197(6):e5325. doi: 10.1002/vetr.5325 (PMC12447684; doi:10.1002/vetr.5325)

Supplementary Information

Supplementary Table 1. Comparison of percentage of herds that were virus testing and positive for BVD, and the within-herd percentage of BVD positive test results for no threshold to the number of tests in order to be defined as virus testing and a minimum number of tests of 60% of the number of breeding cows.

			No minimum	60% of herd tested
Percentage of herds using virus testing in 2023	Beef		48.1%	39.4%
	Dairy		53.6%	41.7%
Percentage of herds that were BVD positive in 2023	Beef	Total	7.5%	8.6%
		Virus	2.0%	2.4%
	Dairy	Total	12.9%	14.5%
		Virus	14.0%	18.7%
Mean percentage of tests that were positive for BVD in virus testing herds in 2023	All herds		0.3%	0.3%
	BVD positive herds		1.6%	1.5%

Supplementary Figure 1. A density plot of the number of individual virus tests submitted from each farm as a proportion of herd size from 4,836 herd-year combinations from 2016 – 2023.

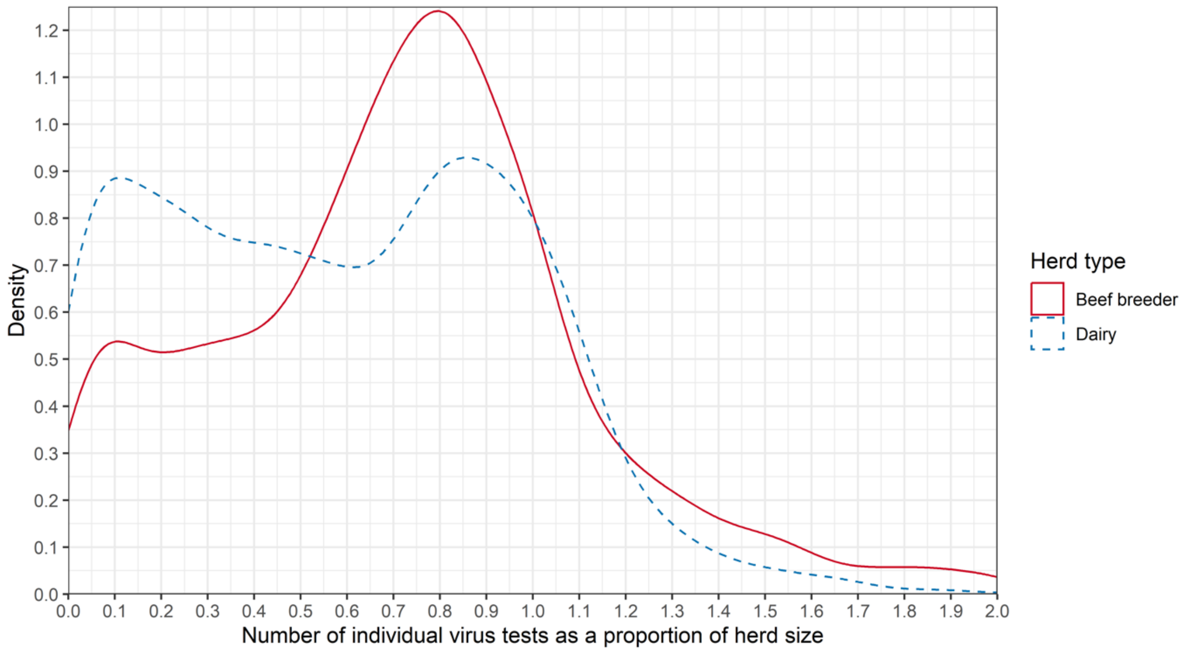

Supplementary Figure 2: The percentage (point) and 95% confidence intervals (error bars) of herds submitting tests to BVDFree England that had at least one positive test result each year from 2016 to 2023 for herds using antibody testing, virus testing, or either testing regime.

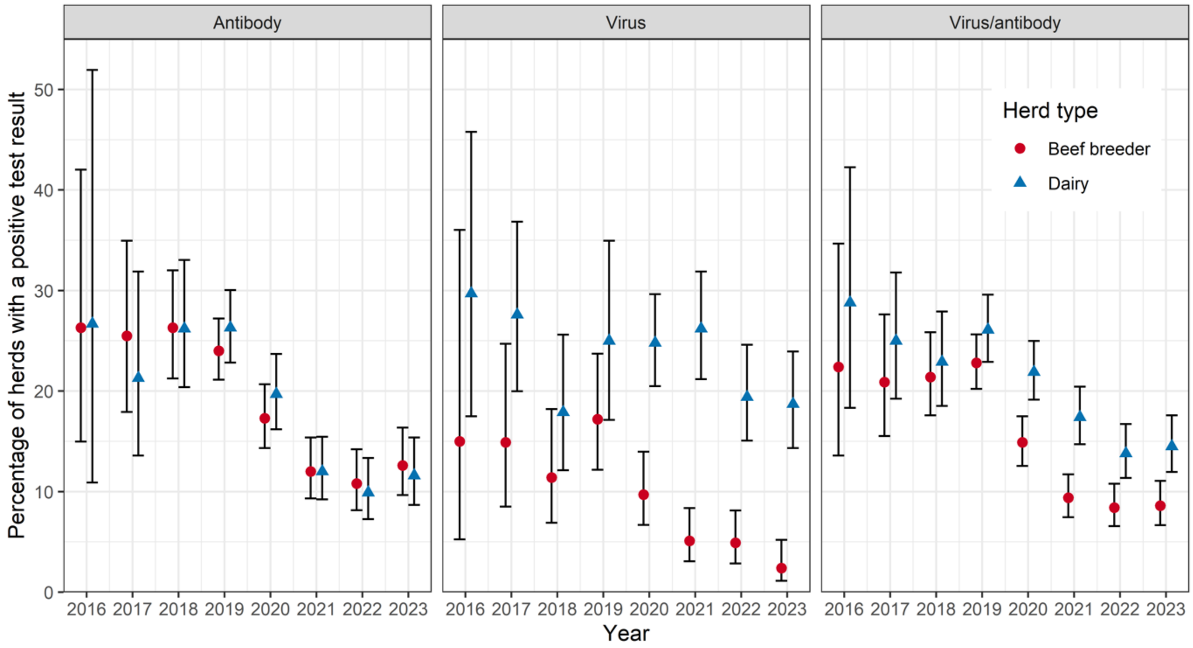

Supplementary Figure 3: The percentage (point) and 95% confidence intervals (error bars) of virus tests that were positive in virus testing herds that submitted tests to BVDFree England between 2016 and 2023.

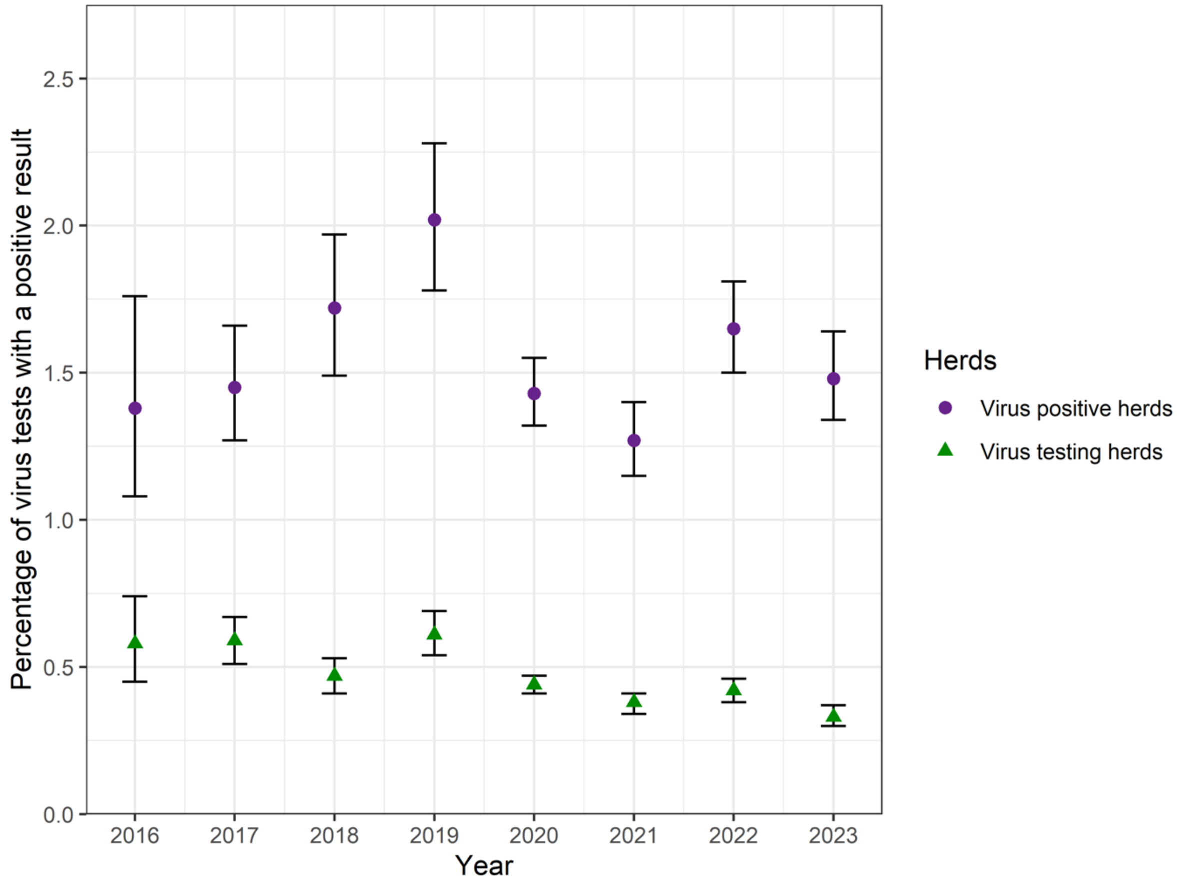

Supplementary Figure 4. Decile plot of the fit of Model 1, a binomial mixed effects model for the odds of a herd having at least one positive bovine viral diarrhoea test result in year 1 explained by the number of consecutive years testing (fixed effect) and herd (random effect) for 1,799 herds that used a testing regime (either virus or antibody) for at least two consecutive years between 2016 and 2023.

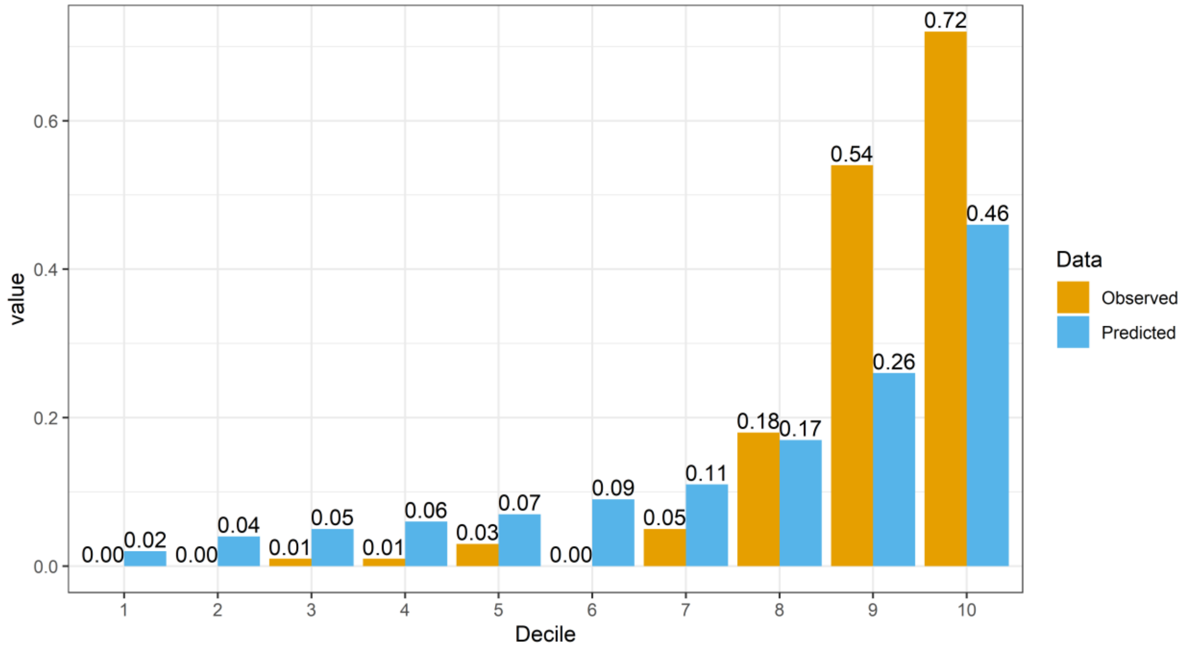

Supplementary Figure 5. Decile plot of the fit of Model 2, a proportional binomial mixed effects model for the odds of herds having at least one positive bovine viral diarrhoea test result in year 1 explained by the number of consecutive years testing (fixed effect) and herd (random effect) for 599 herds that used the testing regime for at least two consecutive years between 2016 and 2023.

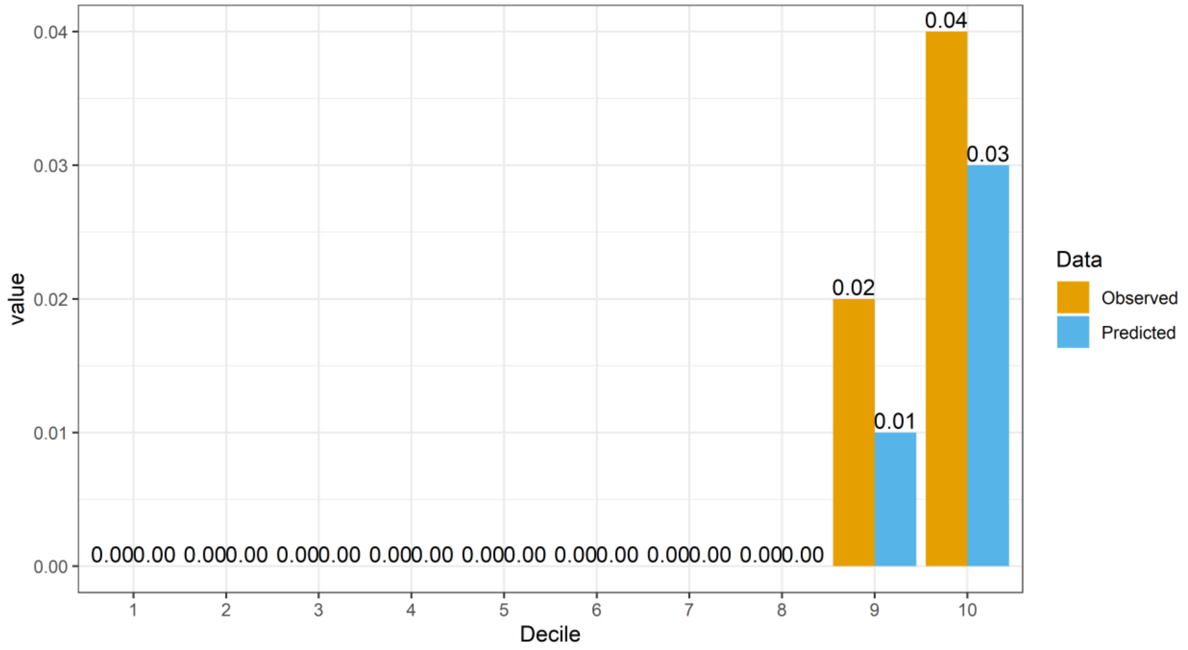

Supplement: Supplementary file 1 — Supporting Information [file VETR-197-e5325-s002.pdf]
