# Supplementary material for: Descriptive analysis of national bovine viral diarrhoea test data in England (2016–2023)
Source: Vet Rec. 2025 May 30;197(6):e5325. doi: 10.1002/vetr.5325 (PMC12447684; doi:10.1002/vetr.5325)
Supplement: Supplementary file 2 — Supporting Information [file VETR-197-e5325-s001.pdf]

```
### BVDFree data analysis ###
### Dataset up to 21/02/2024
### R version: 4.3.1
### Date: 22/07/24

#### Load packages ####
library(plyr)
library(tidyverse)
library(lubridate)
library(reshape2)
library(maps)
library(PostcodesioR) # Calculating regions from postcodes
library(rgdal) # Read in the shapefile
library(rgeos) # For calculating region centroids
library(broom) # Reshape shapefile for ggplot2
library(lme4) # Multilevel models
library(dplyr) # For creating summaries by group
library(ggalluvial) # Alluvial plots

#### Load data ####

# BVDFree dataset
Data <- read.csv("./bvdfree-full-results.csv", fileEncoding="UTF-8-BOM")

# Country shapefile - from Open Geography Portal, Office for National Statistics
Eng_shapefile <- readOGR(dsn="./Countries_(December_2020)_UK_BFE", layer="Countries_(December_2020)_UK_BFE")
Eng_shapefile <- spTransform(Eng_shapefile, CRS("+proj=longlat +ellps=WGS84 +datum=WGS84"))
Eng_mapdata <- tidy(Eng_shapefile) #This might take a few minutes

# UK regions shapefile - from Open Geography Portal, Office for National Statistics
shapefile <- readOGR(dsn="./Regions_(December_2020)_EN_BFE", layer="Regions_(December_2020)_EN_BFE")
shapefile <- spTransform(shapefile, CRS("+proj=longlat +ellps=WGS84 +datum=WGS84"))
mapdata <- tidy(shapefile)

# Add centroids for each region for annotating maps later
region_centres <- SpatialPointsDataFrame(gCentroid(shapefile, byid=TRUE), shapefile@data, match.ID=FALSE)
region_centres <- data.frame(region_centres[,c("RGN20NM", "LONG", "LAT")])
region_centres <- data.frame(region_centres[,1:3])
colnames(region_centres) <- c("region", "region_long", "region_lat")
# Modify centroids for clearer display of labels
# Move NW centroid SW
region_centres$region_long[region_centres$region=="North East"] <- region_centres$region_long[region_centres$region=="North East"] - 0.25
region_centres$region_lat[region_centres$region=="North East"] <- region_centres$region_lat[region_centres$region=="North East"] - 0.25
# Move SW centroid N
region_centres$region_lat[region_centres$region=="South West"] <- region_centres$region_lat[region_centres$region=="South West"] + 0.125
# Move London centroid E
region_centres$region_long[region_centres$region=="London"] <- region_centres$region_long[region_centres$region=="London"] + 0.125
# Move Yorks centroid NE
region_centres$region_long[region_centres$region=="Yorkshire and The Humber"] <- region_centres$region_long[region_centres$region=="Yorkshire and The Humber"] + 0.125
```

```
region_centres$region_lat[region_centres$region=="Yorkshire and The Humber"] <- region_centres$region_lat[region_centres$region=="Yorkshire and The Humber"] + 0.125
# Move E mids centroid NE
region_centres$region_long[region_centres$region=="East Midlands"] <- region_centres$region_long[region_centres$region=="East Midlands"] + 0.125
region_centres$region_lat[region_centres$region=="East Midlands"] <- region_centres$region_lat[region_centres$region=="East Midlands"] + 0.125
# Move SE centroid SE
region_centres$region_long[region_centres$region=="South East"] <- region_centres$region_long[region_centres$region=="South East"] + 0.25
region_centres$region_lat[region_centres$region=="South East"] <- region_centres$region_lat[region_centres$region=="South East"] - 0.25

#### Data preparation ####

# Make empty, NULL or ## cells in dataframe NA
Data <- Data %>% apply(2, function(x){na_if(x, "")}) %>%
  apply(2, function(x){na_if(x, "##")}) %>%
  apply(2, function(x){na_if(x, "NULL")}) %>%
  data.frame()

## Make all categorical columns factor
Factor_columns <- c("test_holding_number","postal_code","farm_type","analysis_type","test_type","sample_type","pooled_sample","result")
Data[,Factor_columns] <- lapply(Data[,Factor_columns], as.factor)

## Farm type

# Split farm_type into separate columns
Data$beef_breeder <- grepl("beefbreeder", Data$farm_type, fixed = TRUE) %>%
  ifelse("1", "0") %>%
  as.factor()
Data$beef_finisher <- grepl("beeffinisher", Data$farm_type, fixed = TRUE) %>%
  ifelse("1", "0") %>%
  as.factor()
Data$calf_rearer <- grepl("calf", Data$farm_type, fixed = TRUE) %>%
  ifelse("1", "0") %>%
  as.factor()
Data$dairy <- grepl("dairy", Data$farm_type, fixed = TRUE) %>%
  ifelse("1", "0") %>%
  as.factor()
Data$beef_breeder[Data$beef_breeder!="1"&Data$beef_finisher!="1"&Data$calf_rearer!="1"&Data$dairy!="1"] <- NA
Data$beef_finisher[is.na(Data$beef_breeder)] <- NA
Data$calf_rearer[is.na(Data$beef_breeder)] <- NA
Data$dairy[is.na(Data$beef_breeder)] <- NA
Data$farm_type <- NULL

# Make beef breeder / dairy column for non-mixed herds
Data$beef_dairy <- NA
Data$beef_dairy[Data$beef_breeder=="1" & Data$beef_finisher=="0" & Data$dairy=="0" & Data$calf_rearer=="0"] <- "Beef breeder"
Data$beef_dairy[Data$beef_breeder=="0" & Data$beef_finisher=="0" & Data$dairy=="1" & Data$calf_rearer=="0"] <- "Dairy"
Data$beef_dairy <- as.factor(Data$beef_dairy)

## Herd size
```

```
# Convert text to numbers
Data$herd_size[Data$herd_size=="approx 35"] <- "35"
Data$herd_size[Data$herd_size=="~80"] <- "80"
# Make herd_size numeric
Data$herd_size <- Data$herd_size %>%
  as.character() %>%
  as.numeric()
# Remove beef breeder and dairy cows with 0 cows
Data$herd_size[Data$herd_size==0 & Data$beef_breeder=="1"] <- NA
Data$herd_size[Data$herd_size==0 & Data$dairy=="1"] <- NA

## Dates

# Make all date columns dates
# dmy columns
ymd_cols <- c("sample_date", "sample_analysis_date")
Data[,ymd_cols] <- lapply(Data[,ymd_cols], ymd)
# dmy_hm column
Data$updated_at <- ymd_hms(Data$updated_at)

# Remove samples before 2016 & after 2023
Data <- subset(Data, sample_date>"2015-12-31")
Data <- subset(Data, sample_date<"2024-01-01")

# Remove samples where sample_date > uploaded_at
Data <- subset(Data, (Data$updated_at >= Data$sample_date))

# Add column of sample year
Data$sample_year <- Data$sample_date %>% year() %>% as.factor

## Test type

# Reorder sample_type
levels(Data$sample_type) <- list("Tissue" = "Tissue", "Blood" = "Blood", "Milk" = "Milk")

# Amalgamate antigen and antigen/virus analysis_type
levels(Data$analysis_type) <- list("BVDAb" = "BVDAb", "BVDAg/v" = c("BVDAg", "BVDv"))

# Make specific columns for each test type
Data$individual_antigen <- 0
Data$individual_antibody <- 0
Data$individual_tissue_antigen <- 0
Data$individual_blood_antigen <- 0
Data$pooled_blood_antigen <- 0
Data$pooled_milk_antigen <- 0
Data$pooled_milk_antibody <- 0
Data$individual_antigen[Data$pooled_sample=="0" & Data$analysis_type=="BVDAg/v"] <- 1
Data$individual_antibody[Data$pooled_sample=="0" & Data$analysis_type=="BVDAb"] <- 1
Data$individual_tissue_antigen[Data$pooled_sample=="0" & Data$analysis_type=="BVDAg/v" & Data$sample_type=="Tissue"] <- 1
```

```
Data$individual_blood_antigen[Data$pooled_sample=="0" & Data$analysis_type=="BVDAg/v" & Data$sample_type=="Blood"] <- 1
Data$pooled_blood_antigen[Data$pooled_sample=="1" & Data$sample_type=="Blood" & Data$analysis_type=="BVDAg/v"] <- 1
Data$pooled_milk_antibody[Data$pooled_sample=="1" & Data$sample_type=="Milk" & Data$analysis_type=="BVDAb"] <- 1
Data$pooled_milk_antigen[Data$pooled_sample=="1" & Data$sample_type=="Milk" & Data$analysis_type=="BVDAg/v"] <- 1

## Test results

# Reorder result levels
Data$result <- as.factor(Data$result)
levels(Data$result) <- list("High Positive" = "High Positive", "Positive" = "Positive", "Low Positive" = "Low Positive", "Suspect" = "Suspect", "Inconclusive" = "Inconclusive", "Negative" = "Negative", "Invalid" = "Invalid", "Unknown" =
"Unknown", "No sample" = "No Sample")

# Simplify results
Data$simple_result <- Data$result
levels(Data$simple_result) <- list("Positive" = c("High Positive", "Positive", "Low Positive"), "Negative" = "Negative", "No result" = c("Suspect", "Inconclusive", "Invalid", "Unknown", "No sample"))

# Make a column of overall antigen test result
Data$antigen_result <- NA
Data$antigen_result[Data$simple_result=="Positive" & Data$analysis_type=="BVDAg/v"] <- 1
Data$antigen_result[Data$simple_result!="Positive"] <- 0
Data$antigen_result[Data$simple_result=="No result"|Data$analysis_type=="BVDAb"] <- NA

# Make column of individual antigen result
Data$antigen_result_individual <- NA
Data$antigen_result_individual[Data$simple_result=="Positive" & Data$analysis_type=="BVDAg/v"] <- 1
Data$antigen_result_individual[Data$simple_result!="Positive"] <- 0
Data$antigen_result_individual[Data$simple_result=="No result"|Data$analysis_type=="BVDAb"|Data$pooled_sample=="1"] <- NA

# Make columns of pooled & individual antibody test results
Data$antibody_result_individual <- NA
Data$antibody_result_individual[Data$simple_result=="Positive" & Data$analysis_type=="BVDAb"] <- 1
Data$antibody_result_individual[Data$simple_result!="Positive"] <- 0
Data$antibody_result_individual[Data$simple_result=="No result"|Data$analysis_type=="BVDAg/v"|Data$pooled_sample=="1"] <- NA

Data$antibody_result_pooled <- NA
Data$antibody_result_pooled[Data$simple_result=="Positive" & Data$analysis_type=="BVDAb"] <- 1
Data$antibody_result_pooled[Data$simple_result!="Positive"] <- 0
Data$antibody_result_pooled[Data$simple_result=="No result"|Data$analysis_type=="BVDAg/v"|Data$pooled_sample=="0"] <- NA

# Remove tests with no holding number
Data <- subset(Data, !is.na(test_holding_number))

# Remove levels that are no longer used
Data <- droplevels(Data)

#### Other datasets ####

#### Make dataset for each year
Data_by_yr_list <- list()
```

```
for(i in 2016:2023){
  Data_by_yr_list[[i-2015]] <- subset(Data, year(sample_date)==i) %>%
    droplevels()
  names(Data_by_yr_list)[i-2015] <- paste("Data", i, sep="")
}

#### Create herd-level datasets
Herd_data_by_yr_list <- Data_by_yr_list
# Variables of interest for herd-level dataset
Herd_data_variables <-
c("test_holding_number","postal_code","herd_size","practice_id","sample_year","beef_breeder","beef_finisher","calf_rearer","dairy","beef_dairy","individual_antigen","individual_tissue_antigen","individual_blood_antigen","individual
_antibody","pooled_blood_antigen","pooled_milk_antigen","pooled_milk_antibody","antigen_result", "n_antigen_tests_pos", "n_antigen_tests", "prop_antigen_tests_pos")
for(i in 1:length(Herd_data_by_yr_list)){
  # Summarise herd test and results by year
  Herd_data_by_yr_list[[i]] <- Herd_data_by_yr_list[[i]] %>% group_by(test_holding_number) %>%
    mutate(individual_antigen = sum(individual_antigen),
           individual_antibody = sum(individual_antibody),
           pooled_blood_antigen = sum(pooled_blood_antigen),
           pooled_milk_antigen = sum(pooled_milk_antigen),
           pooled_milk_antibody = sum(pooled_milk_antibody),
           antigen_result = antigen_result %>% max(na.rm=T),
           n_antigen_tests_pos = sum(antigen_result_individual, na.rm = T),
           n_antigen_tests = sum(antigen_result_individual == 0, na.rm = T),
           prop_antigen_tests_pos = mean(antigen_result_individual, na.rm = T))
  # Retain only the first row for each herd
  Herd_data_by_yr_list[[i]] <- Herd_data_by_yr_list[[i]][!duplicated(Herd_data_by_yr_list[[i]]$test_holding_number), ]
  # Retain only variables of interest
  Herd_data_by_yr_list[[i]] <- Herd_data_by_yr_list[[i]][,Herd_data_variables]
}
rm(Herd_data_variables)
# Concatenate list of dataframes into 1
Herd_data <- ldply(Herd_data_by_yr_list, rbind)[,-1]
Herd_data <- Herd_data %>% subset(!is.na(test_holding_number))

# Add columns for number of dates and occasions individual antibody tests were carried out
Antibody_testing_data <- Data %>% subset(individual_antibody==1) %>%
  group_by(test_holding_number, sample_year) %>%
  # Calculate time between first and last sample
  mutate(individual_antibody_date_range = (max(sample_date) - min(sample_date)),
         # Count number of dates
         individual_antibody_date_number = nlevels(droplevels(as.factor(sample_date))),
         # Select overall herd result
         individual_antibody_result = antibody_result_individual %>% max(na.rm=T)) %>%
  ungroup()
# Convert "-Inf" to NA for herd/years with no positive/negative result for the tests
Antibody_testing_data$individual_antibody_result[Antibody_testing_data$individual_antibody_result=="-Inf"] <- NA
# Retain only one row per herd per year
Antibody_testing_data <- Antibody_testing_data[!duplicated(cbind(Antibody_testing_data$test_holding_number, Antibody_testing_data$sample_year)), ]
# Add columns to add to Herd_data
```

```
Antibody_testing_data <- Antibody_testing_data[,c("test_holding_number","sample_year","individual_antibody_date_range","individual_antibody_date_number","individual_antibody_result")]
Herd_data <- left_join(Herd_data, Antibody_testing_data, by=c("test_holding_number","sample_year"))

# Add columns for the number of dates and date range that individual antigen tests were carried out
Antigen_testing_data <- Data %>% subset(individual_antigen==1) %>%
  group_by(test_holding_number, sample_year) %>%
  # Calculate time between first & last sample
  mutate(individual_antigen_date_range = (max(sample_date) - min(sample_date)),
         # Count number of dates
         individual_antigen_date_number = nlevels(droplevels(as.factor(sample_date))),
         # Select overall herd result
         individual_antigen_result = antigen_result_individual %>% max(na.rm=T)) %>%
  ungroup()
# Convert "-Inf" to NA for herd/years with no positive/negative result for the tests
Antigen_testing_data$individual_antigen_result[Antigen_testing_data$individual_antigen_result=="-Inf"] <- NA
# Retain only one row per herd per year
Antigen_testing_data <- Antigen_testing_data[!duplicated(cbind(Antigen_testing_data$test_holding_number, Antigen_testing_data$sample_year)), ]
# Add columns to add to Herd_data
Antigen_testing_data <- Antigen_testing_data[,c("test_holding_number","sample_year","individual_antigen_date_range","individual_antigen_date_number","individual_antigen_result")]
Herd_data <- left_join(Herd_data, Antigen_testing_data, by=c("test_holding_number","sample_year"))

# Add columns for the number of pooled milk samples & results
# Antigen
Pooled_milk_antigen_data <- Data %>% subset(pooled_milk_antigen==1) %>%
  group_by(test_holding_number, sample_year) %>%
  # Count number of tests
  mutate(pooled_milk_antigen_number = sum(pooled_milk_antigen),
         # Select overall herd result
         pooled_milk_antigen_result = antigen_result %>% max(na.rm=T)) %>%
  ungroup()
# Convert "-Inf" to NA for herd/years with no positive/negative result for the tests
Pooled_milk_antigen_data$pooled_milk_antigen_result[Pooled_milk_antigen_data$pooled_milk_antigen_result=="-Inf"] <- NA
# Retain only one row per herd per year
Pooled_milk_antigen_data <- Pooled_milk_antigen_data[!duplicated(cbind(Pooled_milk_antigen_data$test_holding_number, Pooled_milk_antigen_data$sample_year)), ]
# Add new columns to Herd_data
Pooled_milk_antigen_data <- Pooled_milk_antigen_data[,c("test_holding_number","sample_year","pooled_milk_antigen_number","pooled_milk_antigen_result")]
Herd_data <- left_join(Herd_data, Pooled_milk_antigen_data, by=c("test_holding_number","sample_year"))

# Antibody
Pooled_milk_antibody_data <- Data %>% subset(pooled_milk_antibody==1) %>%
  group_by(test_holding_number, sample_year) %>%
  # Count number of tests
  mutate(pooled_milk_antibody_number = sum(pooled_milk_antibody),
         # Select overall herd result
         pooled_milk_antibody_result = antibody_result_pooled %>% max(na.rm=T)) %>%
  ungroup()
# Retain only one row per herd per year
Pooled_milk_antibody_data <- Pooled_milk_antibody_data[!duplicated(cbind(Pooled_milk_antibody_data$test_holding_number, Pooled_milk_antibody_data$sample_year)), ]
# Add columns to Herd_data
```

```
Pooled_milk_antibody_data <- Pooled_milk_antibody_data[,c("test_holding_number","sample_year","pooled_milk_antibody_number","pooled_milk_antibody_result")]
Herd_data <- left_join(Herd_data, Pooled_milk_antibody_data, by=c("test_holding_number","sample_year"))

# Add a column for whether a herd antigen/antibody testing
Herd_data$test_regime <- NA
Herd_data$test_regime[!is.na(Herd_data$individual_antibody_result) & Herd_data$individual_antibody>4] <- "Antibody"
Herd_data$test_regime[!is.na(Herd_data$individual_antigen_result) & Herd_data$individual_antibody==0 & Herd_data$pooled_blood_antigen==0 & Herd_data$pooled_milk_antigen==0 & Herd_data$pooled_milk_antibody==0] <-
"Antigen"
Herd_data$test_regime <- as.factor(Herd_data$test_regime)

# Add column for test results from either individual antigen testing or individual antibody testing
y <- NULL
for(i in 1:nrow(Herd_data)){
  x <- ifelse((Herd_data[i,]$test_regime=="Antigen"), Herd_data[i,]$individual_antigen_result,
            ifelse(Herd_data[i,]$test_regime=="Antibody", Herd_data[i,]$individual_antibody_result, NA))
  y <- c(y, x)
}
Herd_data$herd_result <- y

Herd_data$test_regime2 <- Herd_data$test_regime
Herd_data$test_regime2[Herd_data$test_regime=="Antigen" & Herd_data$individual_antigen/Herd_data$herd_size<0.6] <- NA

# Add a column of number of years each herd has submitted tests
Herd_data$engagement <- NA
for(i in 1:nrow(Herd_data)){
  Herd_data$engagement[i] <- nrow(subset(Herd_data[1:i,], test_holding_number==Herd_data[i,]$test_holding_number))
}
Herd_data$engagement[Herd_data$engagement=="0"] <- NA

# Add latitude, longitude & regions of postcodes - this take a while
# Find information for each postcode
Postcode_data <- NULL
for(i in levels(Herd_data$postal_code)){
  a <- postcode_lookup(i)
  b <- c(i, a$longitude, a$latitude, a$region)
  Postcode_data <- rbind(Postcode_data, b)
}
colnames(Postcode_data) <- c("postal_code", "longitude", "latitude", "region")
Postcode_data <- as.data.frame(Postcode_data)

# Left_join Postcode_data to Herd_data
Herd_data <- Herd_data %>% left_join(Postcode_data)

# Make latitude & longitude numeric
Herd_data$latitude <- as.numeric(Herd_data$latitude)
Herd_data$longitude <- as.numeric(Herd_data$longitude)

# Make region factor
Herd_data$region <- as.factor(Herd_data$region)
```

```
## Replace -Inf from calculations with NA
Herd_data[Herd_data==-Inf] <- NA

# Remove empty levels
Herd_data <- droplevels(Herd_data)

## Add column for number of consecutive years testing
Consec_yrs_vector <- NULL
Consec_yrs_data <- Herd_data
Consec_yrs_data$sample_year <- Consec_yrs_data$sample_year %>%
  as.character() %>%
  as.numeric()
Consec_yrs_data <- for(i in rownames(Consec_yrs_data)){
  holding <- Consec_yrs_data[i,]$test_holding_number
  year <- Consec_yrs_data[i,]$sample_year
  Temp_data <- Consec_yrs_data %>% subset(test_holding_number==holding & sample_year <= year)
  Temp_data <- Temp_data %>%
    arrange(-sample_year) # Order rows by year, with most recent at top
  X <- split(Temp_data$sample_year, cumsum(c(-1, diff(Temp_data$sample_year) != -1))) # Split data into separate datasets for each set of consecutive years
  X <- length(X$`-1`)
  Consec_yrs_vector <- rbind(Consec_yrs_vector, c(i, X))
}
rm(Consec_yrs_data, Temp_data, holding, year)

Consec_yrs_vector <- as.data.frame(Consec_yrs_vector)
colnames(Consec_yrs_vector) <- c("Row_indices", "Consec_yrs")

# Combine with Herd_data
Herd_data$Row_indices <- rownames(Herd_data)
Herd_data <- merge(Herd_data, Consec_yrs_vector, by="Row_indices", all = TRUE)

#### Recruited herds dataset
Recruited_data <- subset(Herd_data, engagement==1)
# Remove empty levels
Recruited_data <- droplevels(Recruited_data)

#### Herd descriptive statistics ####

### Number of holdings submitting tests per year

## Plot number of herds testing and recruited each year - Figure 1
Plot_data <- rbind(cbind(Herd_data$sample_year, "Tested"), cbind(Recruited_data$sample_year, "Recruited"))
Plot_data <- as.data.frame(Plot_data)
colnames(Plot_data) <- c("Year", "Variable")
Plot_data$Year <- as.factor(Plot_data$Year)
levels(Plot_data$Year) <- list("2016" = "1", "2017" = "2", "2018" = "3", "2019" = "4", "2020" = "5", "2021" = "6", "2022" = "7", "2023" = "8")
png(filename="No_of_holdings_submitting_tests_and_recruited_per_year.png", res=600, width=4000, height=2000)
ggplot(Plot_data, aes(x = Year, fill = Variable)) +
```

```
  geom_bar(position = "dodge") +
  scale_y_continuous(expand = c(0,0), limits = c(0, 4400)) +
  scale_fill_manual(values = c("#CA0020", "#F4A582")) +
  theme_bw() +
  labs(y = "Number of holdings")
dev.off()

# Number of herds recruited & submitting each year
table(Plot_data$Year, Plot_data$Variable)

# Total number of herds
nlevels(Data$test_holding_number)

### Herd sizes engaged in scheme
## Dairy
Dairy_herd_size_testing <- aggregate(herd_size ~ sample_year, subset(Herd_data, dairy==1 & beef_breeder==0 & beef_finisher==0 & calf_rearer==0), median)
Dairy_herd_size_testing
write.csv(Dairy_herd_size_testing, "Median_dairy_herd_size_by_year.csv", row.names = FALSE)
## Beef breeder
Beef_herd_size_testing <- aggregate(herd_size ~ sample_year, subset(Herd_data, dairy==0 & beef_breeder==1 & beef_finisher==0 & calf_rearer==0), median)
Beef_herd_size_testing
write.csv(Beef_herd_size_testing, "Median_beef_herd_size_by_year.csv", row.names = FALSE)

### Types of holdings submitting to scheme each year
## Number of herds which have given their herd type
Known_herd_type_n <- nlevels(droplevels(subset(Herd_data, !is.na(beef_breeder))$test_holding_number))
# Beef breeder n and proportion
nlevels(droplevels(subset(Herd_data, beef_breeder==1)$test_holding_number))
nlevels(droplevels(subset(Herd_data, beef_breeder==1)$test_holding_number)) / Known_herd_type_n
# Dairy n and proportion
nlevels(droplevels(subset(Herd_data, dairy==1)$test_holding_number))
nlevels(droplevels(subset(Herd_data, dairy==1)$test_holding_number)) / Known_herd_type_n
# Beef finisher n and proportion
nlevels(droplevels(subset(Herd_data, beef_finisher==1)$test_holding_number))
nlevels(droplevels(subset(Herd_data, beef_finisher==1)$test_holding_number)) / Known_herd_type_n
# Calf rearer n and proportion
nlevels(droplevels(subset(Herd_data, calf_rearer==1)$test_holding_number))
nlevels(droplevels(subset(Herd_data, calf_rearer==1)$test_holding_number)) / Known_herd_type_n

## Repeat for 2023
## Number of herds which have given their herd type
Known_herd_type_2023_n <- nlevels(droplevels(subset(Herd_data, !is.na(beef_breeder) & sample_year=="2023")$test_holding_number))
# Beef breeder n and proportion
nlevels(droplevels(subset(Herd_data, beef_breeder==1 & sample_year=="2023")$test_holding_number))
nlevels(droplevels(subset(Herd_data, beef_breeder==1 & sample_year=="2023")$test_holding_number)) / Known_herd_type_2023_n
# Dairy n and proportion
nlevels(droplevels(subset(Herd_data, dairy==1 & sample_year=="2023")$test_holding_number))
nlevels(droplevels(subset(Herd_data, dairy==1 & sample_year=="2023")$test_holding_number)) / Known_herd_type_2023_n
# Beef breeder & dairy n and proportion
```

```
nlevels(droplevels(subset(Herd_data, beef_breeder==1 & dairy==1 & sample_year=="2023")$test_holding_number))
nlevels(droplevels(subset(Herd_data, beef_breeder==1 & dairy==1 & sample_year=="2023")$test_holding_number)) / Known_herd_type_2023_n
# No dairy or beef breeding cows n and proportion
nlevels(droplevels(subset(Herd_data, beef_breeder==0 & dairy==0 & sample_year=="2023")$test_holding_number))
nlevels(droplevels(subset(Herd_data, beef_breeder==0 & dairy==0 & sample_year=="2023")$test_holding_number)) / Known_herd_type_2023_n

## How many herds are not mixed?
# N
single_herd_type_n <- nlevels(droplevels(subset(Herd_data,
(as.numeric(as.character(beef_breeder))+as.numeric(as.character(beef_finisher))+as.numeric(as.character(calf_rearer))+as.numeric(as.character(dairy)))==1)$test_holding_number))
# Proportion
nlevels(droplevels(subset(Herd_data, (as.numeric(as.character(beef_breeder))+as.numeric(as.character(beef_finisher))+as.numeric(as.character(calf_rearer))+as.numeric(as.character(dairy)))==1)$test_holding_number)) /
Known_herd_type_n
## How many of each herd type are not mixed herd type
# Beef breeder n and proportion
nlevels(droplevels(subset(Herd_data, as.numeric(as.character(beef_breeder))==1 & as.numeric(as.character(beef_finisher))==0 & as.numeric(as.character(calf_rearer))==0 & as.numeric(as.character(dairy))==0)$test_holding_number))
nlevels(droplevels(subset(Herd_data, as.numeric(as.character(beef_breeder))==1 & as.numeric(as.character(beef_finisher))==0 & as.numeric(as.character(calf_rearer))==0 & as.numeric(as.character(dairy))==0)$test_holding_number)) /
single_herd_type_n
# Dairy n and proportion
nlevels(droplevels(subset(Herd_data, as.numeric(as.character(beef_breeder))==0 & as.numeric(as.character(beef_finisher))==0 & as.numeric(as.character(calf_rearer))==0 & as.numeric(as.character(dairy))==1)$test_holding_number))
nlevels(droplevels(subset(Herd_data, as.numeric(as.character(beef_breeder))==0 & as.numeric(as.character(beef_finisher))==0 & as.numeric(as.character(calf_rearer))==0 & as.numeric(as.character(dairy))==1)$test_holding_number)) /
single_herd_type_n

## Proportion of recruited herds that are dairy (v beef breeders)
Prop_dairy_recruited <- Recruited_data %>%
  mutate(beef_dairy = as.numeric(Recruited_data$beef_dairy) - 1) %>%
  group_by(sample_year) %>%
  dplyr::summarize(mean = mean(beef_dairy, na.rm=T))
Prop_dairy_recruited

## Proportion of herds that are dairy (v beef breeders)
Prop_dairy_testing <- Herd_data %>%
  mutate(beef_dairy = as.numeric(Herd_data$beef_dairy) - 1) %>%
  group_by(sample_year) %>%
  dplyr::summarize(mean = mean(beef_dairy, na.rm=T))
Prop_dairy_testing

# Save dataframe
Prop_dairy <- merge(Prop_dairy_recruited, Prop_dairy_testing, by="sample_year")
colnames(Prop_dairy) <- c("Year", "Recruited", "Testing")
write.csv(Prop_dairy, "Proportion_of_recruited_and_testing_herds_that_are_dairy.csv", row.names = FALSE)

## Region-level data - Figure 2

# Calculate region-level number of herds
Region_data <- Herd_data %>%
  arrange(-engagement) %>%
  distinct(test_holding_number, .keep_all = TRUE)
```

```
levels(Region_data$region) <- list("East Midlands" = "East Midlands",
                                   "East of England" = "East of England",
                                   "North East" = "North East",
                                   "North West" = "North West",
                                   "South East" = c("South East", "London"),
                                   "South West" = "South West",
                                   "West Midlands" = "West Midlands",
                                   "Yorkshire and The Humber" = "Yorkshire and The Humber")
levels(Region_data$region) <- c(levels(Region_data$region), "London") # Force London back in for plotting as blank on map
Region_data_all <- Region_data
Region_data_all$beef_dairy <- "All"
Region_data <- rbind(Region_data, Region_data_all)
Region_data$beef_dairy <- relevel(Region_data$beef_dairy, ref = "All")

Region_data <- Region_data %>% group_by(region, beef_dairy, .drop=FALSE) %>%
  summarise(n_herds = nlevels(droplevels(test_holding_number)),
            n_cows = sum(herd_size, na.rm = TRUE),
            mean_engagement = mean(engagement)) %>%
  subset(!is.na(region) & !is.na(beef_dairy))

## Load DEFRA cow data
Regional_n_cow_data <- read.csv("./DEFRA_n_cows_per_region_2021.csv", fileEncoding="UTF-8-BOM") # Load DEFRA regional dataset
Regional_n_cow_data <- subset(Regional_n_cow_data, England.2021.Region != "England")
Regional_n_cow_data[,-1] <- lapply(Regional_n_cow_data[,-1], function(x) as.numeric(gsub(",", "", x))) # Remove commas from numbers
Regional_n_cow_data$England.2021.Region <- as.factor(Regional_n_cow_data$England.2021.Region)
levels(Regional_n_cow_data$England.2021.Region) <- list("East Midlands" = "East Midlands",
                                                        "East of England" = "Eastern",
                                                        "North East" = "North East",
                                                        "North West" = "North West and Merseyside",
                                                        "South East" = "South East (incl. London)",
                                                        "South West" = "South West",
                                                        "West Midlands" = "West Midlands",
                                                        "Yorkshire and The Humber" = "Yorkshire and The Humber")
Regional_n_cow_data <- Regional_n_cow_data[,c("England.2021.Region", "Dairy.breeding.herd", "Beef.breeding.herd", "Dairy.female.2yr..no.offspring", "Beef.female.2yr..no.offspring")] # Select only rows of interest
## Attach to Region_data
Region_data <- merge(Region_data, Regional_n_cow_data, by.x = "region", by.y = "England.2021.Region", all = TRUE)
## Calculate regional herd size
Region_data$n.breeding.herd.w.offspring <- 0
Region_data$n.breeding.herd.w.offspring[Region_data$beef_dairy == "All"] <- Region_data$Dairy.breeding.herd[Region_data$beef_dairy == "All"] + Region_data$Beef.breeding.herd[Region_data$beef_dairy == "All"]
Region_data$n.breeding.herd.w.offspring[Region_data$beef_dairy == "Beef breeder"] <- Region_data$Beef.breeding.herd[Region_data$beef_dairy == "Beef breeder"]
Region_data$n.breeding.herd.w.offspring[Region_data$beef_dairy == "Dairy"] <- Region_data$Dairy.breeding.herd[Region_data$beef_dairy == "Dairy"]
Region_data$n.breeding.herd <- 0
Region_data$n.breeding.herd[Region_data$beef_dairy == "All"] <- Region_data$Dairy.breeding.herd[Region_data$beef_dairy == "All"] + Region_data$Beef.breeding.herd[Region_data$beef_dairy == "All"] +
Region_data$Dairy.female.2yr..no.offspring[Region_data$beef_dairy == "All"] + Region_data$Beef.female.2yr..no.offspring[Region_data$beef_dairy == "All"]
Region_data$n.breeding.herd[Region_data$beef_dairy == "Beef breeder"] <- Region_data$Beef.breeding.herd[Region_data$beef_dairy == "Beef breeder"] + Region_data$Beef.female.2yr..no.offspring[Region_data$beef_dairy == "Beef
breeder"]
Region_data$n.breeding.herd[Region_data$beef_dairy == "Dairy"] <- Region_data$Dairy.breeding.herd[Region_data$beef_dairy == "Dairy"] + Region_data$Dairy.female.2yr..no.offspring[Region_data$beef_dairy == "Dairy"]
## Calculate proportion of regional herd in BVDFree scheme
Region_data$Prop_cows_w_offspring <- Region_data$n_cows / Region_data$n.breeding.herd.w.offspring
```

```
Region_data$Prop_cows <- Region_data$n_cows / Region_data$n.breeding.herd
# Rename regions to match mapdata
Region_plot_data <- Region_data
Region_plot_data$id <- NULL
Region_plot_data$id[Region_plot_data$region=="North East"] <- "0"
Region_plot_data$id[Region_plot_data$region=="North West"] <- "1"
Region_plot_data$id[Region_plot_data$region=="Yorkshire and The Humber"] <- "2"
Region_plot_data$id[Region_plot_data$region=="East Midlands"] <- "3"
Region_plot_data$id[Region_plot_data$region=="West Midlands"] <- "4"
Region_plot_data$id[Region_plot_data$region=="East of England"] <- "5"
Region_plot_data$id[Region_plot_data$region=="London"] <- "6"
Region_plot_data$id[Region_plot_data$region=="South East"] <- "7"
Region_plot_data$id[Region_plot_data$region=="South West"] <- "8"

# Add in centroid data
Region_plot_data <- left_join(Region_plot_data, region_centres)

# Join mydata with mapdata
Region_plot_data <- left_join(mapdata, Region_plot_data, by="id")

# Label dataset
Region_plot_data$Dataset <- "a)"

#### Repeat for 2023

## Region-level data

# Calculate region-level number of herds
Region_data_2023 <- Herd_data %>%
  subset(sample_year=="2023") %>%
  arrange(-engagement) %>%
  distinct(test_holding_number, .keep_all = TRUE)

levels(Region_data_2023$region) <- list("East Midlands" = "East Midlands",
                                  "East of England" = "East of England",
                                  "North East" = "North East",
                                  "North West" = "North West",
                                  "South East" = c("South East", "London"),
                                  "South West" = "South West",
                                  "West Midlands" = "West Midlands",
                                  "Yorkshire and The Humber" = "Yorkshire and The Humber")
levels(Region_data_2023$region) <- c(levels(Region_data_2023$region), "London") # Force London back in for plotting as blank on map
Region_data_2023_all <- Region_data_2023
Region_data_2023_all$beef_dairy <- "All"
Region_data_2023 <- rbind(Region_data_2023, Region_data_2023_all)
Region_data_2023$beef_dairy <- relevel(Region_data_2023$beef_dairy, ref = "All")

Region_data_2023 <- Region_data_2023 %>% group_by(region, beef_dairy, .drop=FALSE) %>%
  summarise(n_herds = nlevels(droplevels(test_holding_number)),
```

```
      n_cows = sum(herd_size, na.rm = TRUE),
      mean_engagement = mean(engagement)) %>%
  subset(!is.na(region) & !is.na(beef_dairy))

## Attach DEFRA cow data to Region_data
Region_data_2023 <- merge(Region_data_2023, Regional_n_cow_data, by.x = "region", by.y = "England.2021.Region", all = TRUE)
## Calculate regional herd size
Region_data_2023$n.breeding.herd.w.offspring <- 0
Region_data_2023$n.breeding.herd.w.offspring[Region_data_2023$beef_dairy == "All"] <- Region_data_2023$Dairy.breeding.herd[Region_data_2023$beef_dairy == "All"] +
Region_data_2023$Beef.breeding.herd[Region_data_2023$beef_dairy == "All"]
Region_data_2023$n.breeding.herd.w.offspring[Region_data_2023$beef_dairy == "Beef breeder"] <- Region_data_2023$Beef.breeding.herd[Region_data_2023$beef_dairy == "Beef breeder"]
Region_data_2023$n.breeding.herd.w.offspring[Region_data_2023$beef_dairy == "Dairy"] <- Region_data_2023$Dairy.breeding.herd[Region_data_2023$beef_dairy == "Dairy"]
Region_data_2023$n.breeding.herd <- 0
Region_data_2023$n.breeding.herd[Region_data_2023$beef_dairy == "All"] <- Region_data_2023$Dairy.breeding.herd[Region_data_2023$beef_dairy == "All"] + Region_data_2023$Beef.breeding.herd[Region_data_2023$beef_dairy ==
"All"] + Region_data_2023$Dairy.female.2yr..no.offspring[Region_data_2023$beef_dairy == "All"] + Region_data_2023$Beef.female.2yr..no.offspring[Region_data_2023$beef_dairy == "All"]
Region_data_2023$n.breeding.herd[Region_data_2023$beef_dairy == "Beef breeder"] <- Region_data_2023$Beef.breeding.herd[Region_data_2023$beef_dairy == "Beef breeder"] +
Region_data_2023$Beef.female.2yr..no.offspring[Region_data_2023$beef_dairy == "Beef breeder"]
Region_data_2023$n.breeding.herd[Region_data_2023$beef_dairy == "Dairy"] <- Region_data_2023$Dairy.breeding.herd[Region_data_2023$beef_dairy == "Dairy"] +
Region_data_2023$Dairy.female.2yr..no.offspring[Region_data_2023$beef_dairy == "Dairy"]
## Calculate proportion of regional herd in BVDFree scheme
Region_data_2023$Prop_cows_w_offspring <- Region_data_2023$n_cows / Region_data_2023$n.breeding.herd.w.offspring
Region_data_2023$Prop_cows <- Region_data_2023$n_cows / Region_data_2023$n.breeding.herd
# Rename regions to match mapdata
Region_plot_data_2023 <- Region_data_2023
Region_plot_data_2023$id <- NULL
Region_plot_data_2023$id[Region_plot_data_2023$region=="North East"] <- "0"
Region_plot_data_2023$id[Region_plot_data_2023$region=="North West"] <- "1"
Region_plot_data_2023$id[Region_plot_data_2023$region=="Yorkshire and The Humber"] <- "2"
Region_plot_data_2023$id[Region_plot_data_2023$region=="East Midlands"] <- "3"
Region_plot_data_2023$id[Region_plot_data_2023$region=="West Midlands"] <- "4"
Region_plot_data_2023$id[Region_plot_data_2023$region=="East of England"] <- "5"
Region_plot_data_2023$id[Region_plot_data_2023$region=="London"] <- "6"
Region_plot_data_2023$id[Region_plot_data_2023$region=="South East"] <- "7"
Region_plot_data_2023$id[Region_plot_data_2023$region=="South West"] <- "8"

# Add in centroid data
Region_plot_data_2023 <- left_join(Region_plot_data_2023, region_centres)

# Join mydata with mapdata
Region_plot_data_2023 <- left_join(mapdata, Region_plot_data_2023, by="id")

# Label dataset
Region_plot_data_2023$Dataset <- "b)"

Region_plot_data_2023 <- rbind(subset(Region_plot_data, beef_dairy=="All"), subset(Region_plot_data_2023, beef_dairy=="All"))

# Heatmap of proportion of herd from each region in scheme
png(filename="Proportion_of_regional_breeding_herd_in_BVDFree_all_years_and_2023.png", res=600, width=7500, height=3500)
ggplot(data = Region_plot_data_2023) +
```

```
  geom_polygon(aes(x = long, y = lat, group = group, fill = Prop_cows), color = "Black", size = 0.25) +
  facet_grid(. ~ Dataset) +
  scale_fill_gradient(low = "white", high = "red", limits=c(0,1)) +
  geom_text(aes(label = paste(formatC(round(Prop_cows, 2), format='f', digits=2), sep=""), x = region_long, y = region_lat)) +
  theme_void() +
  theme(legend.position = 'none', strip.text.x = element_text(size = 15), strip.text.y = element_text(size = 15))
dev.off()

#### Testing descriptive statistics ####

### Types of tests used by different types of herd
## Antigen herds
nlevels(droplevels(subset(Herd_data, test_regime2=="Antigen")$test_holding_number))
## Antibody herds
nlevels(droplevels(subset(Herd_data, test_regime2=="Antibody")$test_holding_number))
# N herd-years
summary(Herd_data$test_regime2)

### Plot test regimes used by beef and dairy herds by year

Plot_data <- subset(Herd_data, !is.na(beef_dairy) & !is.na(test_regime2))
levels(Plot_data$test_regime) <- list("Virus"="Antigen", "Antibody"="Antibody")
levels(Plot_data$beef_dairy) <- list("Beef\nbreeder"="Beef breeder", "Dairy"="Dairy")
png(filename="Test_regimes_by_herd_type_per_year_60per_antigen.png", res=600, width=5000, height=2750)
ggplot(Plot_data, aes(x = beef_dairy, fill = test_regime)) +
  facet_grid(. ~ sample_year) +
  geom_bar() +
  labs(x = "Type of herd", y = "Number of herds") +
  scale_fill_discrete(name="Test regime")
dev.off()

## Save plot dataset
Test_regime_proportions <- table(Plot_data$sample_year, Plot_data$test_regime2, Plot_data$beef_dairy) %>%
  as.data.frame() %>%
  pivot_wider(names_from = Var2, values_from = Freq)
colnames(Test_regime_proportions) <- c("Year", "Herd_type", "Antibody_n", "Antigen_n")
Test_regime_proportions$N_herds <- rowSums(Test_regime_proportions[,c("Antibody_n", "Antigen_n")])
Test_regime_proportions$Antibody_prop <- Test_regime_proportions$Antibody_n / Test_regime_proportions$N_herds
Test_regime_proportions$Antigen_prop <- Test_regime_proportions$Antigen_n / Test_regime_proportions$N_herds
write.csv(Test_regime_proportions, "Test_regime_proportions.csv")

# No 60% min antigen tests
Plot_data <- subset(Herd_data, !is.na(beef_dairy) & !is.na(test_regime))
levels(Plot_data$test_regime) <- list("Virus"="Antigen", "Antibody"="Antibody")
levels(Plot_data$beef_dairy) <- list("Beef\nbreeder"="Beef breeder", "Dairy"="Dairy")
png(filename="Test_regimes_by_herd_type_per_year.png", res=600, width=5000, height=2750)
ggplot(Plot_data, aes(x = beef_dairy, fill = test_regime)) +
  facet_grid(. ~ sample_year) +
  geom_bar() +
```

```
  labs(x = "Type of herd", y = "Number of herds") +
  scale_fill_discrete(name="Test regime")
dev.off()

### Plot of number of individual antigen tests per herd - Supplementary Figure 1
# Subset to herds with at least 1 breeding cow in a breeding herd only & individual antigen tests only
Plot_data <- subset(Herd_data, herd_size>0 & test_regime=="Antigen" & !is.na(beef_dairy))
# By herd type
png(filename="Antigen_tests_as_proportion_of_herd_size.png", res=600, width=5000, height=2750)
ggplot(data = subset(Plot_data, !is.na(beef_dairy)), aes(x = individual_antigen/herd_size)) +
  geom_density(aes(colour = beef_dairy, linetype = beef_dairy)) +
  scale_colour_manual(values = c("Beef breeder"="#CA0020", "Dairy"="#0571B0"), name = "Herd type") +
  scale_linetype_manual(values = c("Beef breeder"=1, "Dairy"=2), name = "Herd type") +
  scale_x_continuous(expand = c(0,0), breaks = seq(0, 2, 0.1), limits=c(0,2)) +
  scale_y_continuous(expand = c(0,0), limits = c(0,1.25), breaks = seq(0,1.2,0.1)) +
  labs(x = "Number of individual virus tests as a proportion of herd size", y = "Density") +
  theme_bw()
dev.off()

#### Herd-level test results descriptive statistics ####

### Antigen test results by year, herd type & testing regime with 60% of herd size antigen definition - Supplementary Figure 2

## Individual antigen testing
# Remove herds that were not solely individual antigen test or were mixed herd type
Antigen_plot_data <- subset(Herd_data, !is.na(beef_dairy) & test_regime2=="Antigen")
# Select variables for plot
Antigen_plot_data <- Antigen_plot_data[,c("sample_year","beef_dairy","individual_antigen_result")]
# Make indivdual antigen result a factor
Antigen_plot_data$individual_antigen_result <- as.factor(Antigen_plot_data$individual_antigen_result)
levels(Antigen_plot_data$individual_antigen_result) <- list("Positive"="1", "Negative"="0")
# Make dataset long
Antigen_plot_data <- melt(Antigen_plot_data[,c("sample_year","individual_antigen_result","beef_dairy")])
Antigen_plot_data$value <- 1
# Make dataset wide
Antigen_plot_data <- aggregate(. ~ sample_year + individual_antigen_result + beef_dairy, data = Antigen_plot_data, FUN = sum)
# Make columns giving position and labels of plot labels
pos_vector <- as.vector(by(Antigen_plot_data$value, list(Antigen_plot_data$sample_year, Antigen_plot_data$beef_dairy), sum))
Antigen_plot_data$pos <- c(rep(pos_vector[1:(length(pos_vector)/2)],2), rep(pos_vector[((length(pos_vector)/2)+1):length(pos_vector)],2))
Antigen_plot_data$prop <- formatC((Antigen_plot_data$value * 100) / Antigen_plot_data$pos, format="f", digits = 1) %>%
  as.numeric
# Retain only rows for positive result
Antigen_plot_data <- subset(Antigen_plot_data, individual_antigen_result=="Positive")

LCI <- NULL
UCI <- NULL
for(j in 1:nrow(Antigen_plot_data)){
  y <- round(prop.test(x = Antigen_plot_data[j,]$value, n = Antigen_plot_data[j,]$pos, conf.level=.95, correct=FALSE)$conf.int[1], digits = 4) * 100
  z <- round(prop.test(x = Antigen_plot_data[j,]$value, n = Antigen_plot_data[j,]$pos, conf.level=.95, correct=FALSE)$conf.int[2], digits = 4) * 100
```

```
  LCI <- c(LCI, y)
  UCI <- c(UCI, z)
}

Antigen_plot_data <- cbind(Antigen_plot_data, LCI, UCI)

## Individual antibody testing
# Remove herds that did not have a at least 5 antibody tests or were mixed herd type
Antibody_plot_data <- subset(Herd_data, !is.na(beef_dairy) & test_regime2=="Antibody")
# Select variables for plot
Antibody_plot_data <- Antibody_plot_data[,c("sample_year","beef_dairy","individual_antibody_result")]
# Make indivdual antibody result a factor
Antibody_plot_data$individual_antibody_result <- as.factor(Antibody_plot_data$individual_antibody_result)
levels(Antibody_plot_data$individual_antibody_result) <- list("Positive"="1", "Negative"="0")
# Make dataset long
Antibody_plot_data <- melt(Antibody_plot_data[,c("sample_year","individual_antibody_result","beef_dairy")])
Antibody_plot_data$value <- 1
# Make dataset wide
Antibody_plot_data <- aggregate(. ~ sample_year + individual_antibody_result + beef_dairy, data = Antibody_plot_data, FUN = sum)
# Make columns giving position and labels of plot labels
pos_vector <- as.vector(by(Antibody_plot_data$value, list(Antibody_plot_data$sample_year, Antibody_plot_data$beef_dairy), sum))
Antibody_plot_data$pos <- c(rep(pos_vector[1:(length(pos_vector)/2)],2), rep(pos_vector[((length(pos_vector)/2)+1):length(pos_vector)],2))
Antibody_plot_data$prop <- formatC((Antibody_plot_data$value * 100) / Antibody_plot_data$pos, format="f", digits = 1) %>%
  as.numeric
# Retain only rows for positive result
Antibody_plot_data <- subset(Antibody_plot_data, individual_antibody_result=="Positive")

LCI <- NULL
UCI <- NULL
for(j in 1:nrow(Antibody_plot_data)){
  y <- round(prop.test(x = Antibody_plot_data[j,]$value, n = Antibody_plot_data[j,]$pos, conf.level=.95, correct=FALSE)$conf.int[1], digits = 4) * 100
  z <- round(prop.test(x = Antibody_plot_data[j,]$value, n = Antibody_plot_data[j,]$pos, conf.level=.95, correct=FALSE)$conf.int[2], digits = 4) * 100
  LCI <- c(LCI, y)
  UCI <- c(UCI, z)
}

Antibody_plot_data <- cbind(Antibody_plot_data, LCI, UCI)

## Individual antigen or individual antibody testing
# Remove herds that do not conform to tissue or blood antibody testing or were mixed herd type
Antigen_antibody_plot_data <- subset(Herd_data, !is.na(beef_dairy) & !is.na(herd_result) & !is.na(test_regime2))
# Select variables for plot
Antigen_antibody_plot_data <- Antigen_antibody_plot_data[,c("sample_year","beef_dairy","herd_result")]
# Make result a factor
Antigen_antibody_plot_data$herd_result <- as.factor(Antigen_antibody_plot_data$herd_result)
levels(Antigen_antibody_plot_data$herd_result) <- list("Positive"="1", "Negative"="0")
# Make dataset long
Antigen_antibody_plot_data <- melt(Antigen_antibody_plot_data[,c("sample_year","herd_result","beef_dairy")])
Antigen_antibody_plot_data$value <- 1
```

```
# Make dataset wide
Antigen_antibody_plot_data <- aggregate(. ~ sample_year + herd_result + beef_dairy, data = Antigen_antibody_plot_data, FUN = sum)
# Make columns giving position and labels of plot labels
pos_vector <- as.vector(by(Antigen_antibody_plot_data$value, list(Antigen_antibody_plot_data$sample_year, Antigen_antibody_plot_data$beef_dairy), sum))
Antigen_antibody_plot_data$pos <- c(rep(pos_vector[1:(length(pos_vector)/2)],2), rep(pos_vector[((length(pos_vector)/2)+1):length(pos_vector)],2))
Antigen_antibody_plot_data$prop <- formatC((Antigen_antibody_plot_data$value * 100) / Antigen_antibody_plot_data$pos, format="f", digits = 1) %>%
  as.numeric
# Retain only rows for positive result
Antigen_antibody_plot_data <- subset(Antigen_antibody_plot_data, herd_result=="Positive")

LCI <- NULL
UCI <- NULL
for(j in 1:nrow(Antigen_antibody_plot_data)){
  y <- round(prop.test(x = Antigen_antibody_plot_data[j,]$value, n = Antigen_antibody_plot_data[j,]$pos, conf.level=.95, correct=FALSE)$conf.int[1], digits = 4) * 100
  z <- round(prop.test(x = Antigen_antibody_plot_data[j,]$value, n = Antigen_antibody_plot_data[j,]$pos, conf.level=.95, correct=FALSE)$conf.int[2], digits = 4) * 100
  LCI <- c(LCI, y)
  UCI <- c(UCI, z)
}

Antigen_antibody_plot_data <- cbind(Antigen_antibody_plot_data, LCI, UCI)

# Combine datasets
# Make column to distinguish test type denominator
Antigen_plot_data$tests <- "Antigen"
Antibody_plot_data$tests <- "Antibody"
Antigen_antibody_plot_data$tests <- "Antigen/antibody"
# Remove result column
Antigen_plot_data$individual_antigen_result <- NULL
Antibody_plot_data$individual_antibody_result <- NULL
Antigen_antibody_plot_data$herd_result <- NULL
# Combine all test type dataframes
Plot_data <- rbind(Antigen_plot_data, Antibody_plot_data, Antigen_antibody_plot_data)

## Edit sample_year so that beef and dairy are plotted next to each other
Plot_data$sample_year <- as.numeric(as.character(Plot_data$sample_year))
y <- NULL
for(i in 1:nrow(Plot_data)){
  x <- ifelse(Plot_data[i,]$beef_dairy=="Beef breeder", Plot_data[i,]$sample_year - 0.125, Plot_data[i,]$sample_year + 0.125)
  y <- rbind(y, x)
}
Plot_data$sample_year <- y

# Rename tests to make "Antigen" "Virus"
Plot_data$tests <- as.factor(Plot_data$tests)
levels(Plot_data$tests) <- list("Antibody" = "Antibody", "Virus" = "Antigen", "Virus/antibody" = "Antigen/antibody")

# Plot
png(filename="Herd-level_test_results_60per_virus_testing.png", res=600, width=5000, height=2750)
ggplot(data = Plot_data, aes(x = sample_year, y = prop)) +
```

```
  geom_errorbar(aes(ymax = UCI, ymin = LCI), width = 0.25) +
  geom_point(aes(color = beef_dairy, shape = beef_dairy), size = 2) +
  facet_grid(. ~ tests) +
  scale_colour_manual(values = c("Beef breeder"="#CA0020", "Dairy"="#0571B0"), name = "Herd type") +
  scale_shape_manual(values = c(19, 17), name = "Herd type") +
  scale_y_continuous(limits = c(0,55), breaks = c(0,10,20,30,40,50), expand = c(0, 0)) +
  scale_x_continuous(limits = c(2015.5,2023.5), breaks = c(2016,2017,2018,2019,2020,2021,2022,2023), expand = c(0, 0)) +
  labs(x = "Year", y = "Percentage of herds with a positive test result") +
  theme_bw() +
  theme(legend.position = c(0.9, 0.8))
dev.off()

## No minimum number of tests for antigen testing

## Individual antigen testing
# Remove herds that were not solely individual antigen test or were mixed herd type
Antigen_plot_data_no_min <- subset(Herd_data, !is.na(beef_dairy) & test_regime=="Antigen")
# Select variables for plot
Antigen_plot_data_no_min <- Antigen_plot_data_no_min[,c("sample_year","beef_dairy","individual_antigen_result")]
# Make indivdual antigen result a factor
Antigen_plot_data_no_min$individual_antigen_result <- as.factor(Antigen_plot_data_no_min$individual_antigen_result)
levels(Antigen_plot_data_no_min$individual_antigen_result) <- list("Positive"="1", "Negative"="0")
# Make dataset long
Antigen_plot_data_no_min <- melt(Antigen_plot_data_no_min[,c("sample_year","individual_antigen_result","beef_dairy")])
Antigen_plot_data_no_min$value <- 1
# Make dataset wide
Antigen_plot_data_no_min <- aggregate(. ~ sample_year + individual_antigen_result + beef_dairy, data = Antigen_plot_data_no_min, FUN = sum)
# Make columns giving position and labels of plot labels
pos_vector <- as.vector(by(Antigen_plot_data_no_min$value, list(Antigen_plot_data_no_min$sample_year, Antigen_plot_data_no_min$beef_dairy), sum))
Antigen_plot_data_no_min$pos <- c(rep(pos_vector[1:(length(pos_vector)/2)],2), rep(pos_vector[((length(pos_vector)/2)+1):length(pos_vector)],2))
Antigen_plot_data_no_min$prop <- formatC((Antigen_plot_data_no_min$value * 100) / Antigen_plot_data_no_min$pos, format="f", digits = 1) %>%
  as.numeric
# Retain only rows for positive result
Antigen_plot_data_no_min <- subset(Antigen_plot_data_no_min, individual_antigen_result=="Positive")

LCI <- NULL
UCI <- NULL
for(j in 1:nrow(Antigen_plot_data_no_min)){
  y <- round(prop.test(x = Antigen_plot_data_no_min[j,]$value, n = Antigen_plot_data_no_min[j,]$pos, conf.level=.95, correct=FALSE)$conf.int[1], digits = 4) * 100
  z <- round(prop.test(x = Antigen_plot_data_no_min[j,]$value, n = Antigen_plot_data_no_min[j,]$pos, conf.level=.95, correct=FALSE)$conf.int[2], digits = 4) * 100
  LCI <- c(LCI, y)
  UCI <- c(UCI, z)
}

Antigen_plot_data_no_min <- cbind(Antigen_plot_data_no_min, LCI, UCI)

## Individual antigen or individual antibody testing
# Remove herds that do not conform to tissue or blood antibody testing or were mixed herd type
Antigen_antibody_plot_data_no_min <- subset(Herd_data, !is.na(beef_dairy) & !is.na(herd_result) & !is.na(test_regime))
```

```
# Select variables for plot
Antigen_antibody_plot_data_no_min <- Antigen_antibody_plot_data_no_min[,c("sample_year","beef_dairy","herd_result")]
# Make result a factor
Antigen_antibody_plot_data_no_min$herd_result <- as.factor(Antigen_antibody_plot_data_no_min$herd_result)
levels(Antigen_antibody_plot_data_no_min$herd_result) <- list("Positive"="1", "Negative"="0")
# Make dataset long
Antigen_antibody_plot_data_no_min <- melt(Antigen_antibody_plot_data_no_min[,c("sample_year","herd_result","beef_dairy")])
Antigen_antibody_plot_data_no_min$value <- 1
# Make dataset wide
Antigen_antibody_plot_data_no_min <- aggregate(. ~ sample_year + herd_result + beef_dairy, data = Antigen_antibody_plot_data_no_min, FUN = sum)
# Make columns giving position and labels of plot labels
pos_vector <- as.vector(by(Antigen_antibody_plot_data_no_min$value, list(Antigen_antibody_plot_data_no_min$sample_year, Antigen_antibody_plot_data_no_min$beef_dairy), sum))
Antigen_antibody_plot_data_no_min$pos <- c(rep(pos_vector[1:(length(pos_vector)/2)],2), rep(pos_vector[((length(pos_vector)/2)+1):length(pos_vector)],2))
Antigen_antibody_plot_data_no_min$prop <- formatC((Antigen_antibody_plot_data_no_min$value * 100) / Antigen_antibody_plot_data_no_min$pos, format="f", digits = 1) %>%
  as.numeric
# Retain only rows for positive result
Antigen_antibody_plot_data_no_min <- subset(Antigen_antibody_plot_data_no_min, herd_result=="Positive")

LCI <- NULL
UCI <- NULL
for(j in 1:nrow(Antigen_antibody_plot_data_no_min)){
  y <- round(prop.test(x = Antigen_antibody_plot_data_no_min[j,]$value, n = Antigen_antibody_plot_data_no_min[j,]$pos, conf.level=.95, correct=FALSE)$conf.int[1], digits = 4) * 100
  z <- round(prop.test(x = Antigen_antibody_plot_data_no_min[j,]$value, n = Antigen_antibody_plot_data_no_min[j,]$pos, conf.level=.95, correct=FALSE)$conf.int[2], digits = 4) * 100
  LCI <- c(LCI, y)
  UCI <- c(UCI, z)
}

Antigen_antibody_plot_data_no_min <- cbind(Antigen_antibody_plot_data_no_min, LCI, UCI)

# Combine datasets
# Make column to distinguish test type denominator
Antigen_plot_data_no_min$tests <- "Antigen"
Antigen_antibody_plot_data_no_min$tests <- "Antigen/antibody"
# Remove result column
Antigen_plot_data_no_min$individual_antigen_result <- NULL
Antigen_antibody_plot_data_no_min$herd_result <- NULL
# Combine all test type dataframes
Plot_data <- rbind(Antigen_plot_data_no_min, Antibody_plot_data, Antigen_antibody_plot_data_no_min)

## Edit sample_year so that beef and dairy are plotted next to each other
Plot_data$sample_year <- as.numeric(as.character(Plot_data$sample_year))
y <- NULL
for(i in 1:nrow(Plot_data)){
  x <- ifelse(Plot_data[i,]$beef_dairy=="Beef breeder", Plot_data[i,]$sample_year - 0.125, Plot_data[i,]$sample_year + 0.125)
  y <- rbind(y, x)
}
Plot_data$sample_year <- y

# Rename tests to make "Antigen" "Virus"
```

```
Plot_data$tests <- as.factor(Plot_data$tests)
levels(Plot_data$tests) <- list("Antibody" = "Antibody", "Virus" = "Antigen", "Virus/antibody" = "Antigen/antibody")

# Plot
png(filename="Herd-level_test_results_no_anitgen_min.png", res=600, width=5000, height=2750)
ggplot(data = Plot_data, aes(x = sample_year, y = prop)) +
  geom_errorbar(aes(ymax = UCI, ymin = LCI), width = 0.25) +
  geom_point(aes(color = beef_dairy, shape = beef_dairy), size = 2) +
  facet_grid(. ~ tests) +
  scale_colour_manual(values = c("Beef breeder"="#CA0020", "Dairy"="#0571B0"), name = "Herd type") +
  scale_shape_manual(values = c(19, 17), name = "Herd type") +
  scale_y_continuous(limits = c(0,55), breaks = c(0,10,20,30,40,50), expand = c(0, 0)) +
  scale_x_continuous(limits = c(2015.5,2023.5), breaks = c(2016,2017,2018,2019,2020,2021,2022,2023), expand = c(0, 0)) +
  labs(x = "Year", y = "Percentage of herds with a positive test result") +
  theme_bw() +
  theme(legend.position = c(0.9, 0.8))
dev.off()

### Herd-level test results by number of years testing - Figure 3

for(i in 3:max(Herd_data$engagement)){

  ## Select only first testing bout for each herd
  Plot_data <- Herd_data %>%
    subset(Consec_yrs==engagement & !is.na(test_regime2) & !is.na(beef_dairy))

  ## Select only herds that have i years of engagement
  Plot_data <- Plot_data %>%
    subset(engagement <= i)
  Plot_data$engagement <- as.factor(Plot_data$engagement)

  N_yrs <- table(Plot_data$test_holding_number)
  Plot_data <- Plot_data %>%
    subset(test_holding_number %in% names(N_yrs[N_yrs == i]))
  Plot_data <- droplevels(Plot_data)
  rm(N_yrs)

  ## Individual antigen testing dataset
  # Remove herds that were not solely individual antigen test or were mixed herd type
  Antigen_plot_data <- Plot_data %>%
    subset(test_regime2=="Antigen")
  # Remove herds that did not consecutively antigen test
  N_yrs <- table(Antigen_plot_data$test_holding_number)
  Antigen_plot_data <- Antigen_plot_data %>%
    subset(test_holding_number %in% names(N_yrs[N_yrs == i]))
  Antigen_plot_data <- droplevels(Antigen_plot_data)
  rm(N_yrs)

  # Select variables for plot
```

```
Antigen_plot_data <- Antigen_plot_data[,c("engagement","beef_dairy","individual_antigen_result")]
# Make indivdual antigen result a factor
Antigen_plot_data$individual_antigen_result <- as.factor(Antigen_plot_data$individual_antigen_result)
levels(Antigen_plot_data$individual_antigen_result) <- list("Positive"="1", "Negative"="0")
# Make dataset long
Antigen_plot_data <- melt(Antigen_plot_data[,c("engagement","individual_antigen_result","beef_dairy")])
Antigen_plot_data$value <- 1
# Make dataset wide
Antigen_plot_data <- aggregate(. ~ engagement + individual_antigen_result + beef_dairy, data = Antigen_plot_data, FUN = sum, drop = FALSE)
# Make NA 0
Antigen_plot_data$value[is.na(Antigen_plot_data$value)] <- 0
# Make columns giving position and labels of plot labels
pos_vector <- as.vector(by(Antigen_plot_data$value, list(Antigen_plot_data$engagement, Antigen_plot_data$beef_dairy), sum))
Antigen_plot_data$pos <- c(rep(pos_vector[1:(length(pos_vector)/2)],2), rep(pos_vector[((length(pos_vector)/2)+1):length(pos_vector)],2))
Antigen_plot_data$prop <- formatC((Antigen_plot_data$value * 100) / Antigen_plot_data$pos, format="f", digits = 2) %>%
  as.numeric
# Retain only rows for positive result
Antigen_plot_data <- subset(Antigen_plot_data, individual_antigen_result=="Positive")

## Individual antibody testing
# Remove herds that did not have a at least 5 antibody tests or were mixed herd type
Antibody_plot_data <- subset(Plot_data, test_regime2=="Antibody")
# Remove herds that did not consecutively Antibody test
N_yrs <- table(Antibody_plot_data$test_holding_number)
Antibody_plot_data <- Antibody_plot_data %>%
  subset(test_holding_number %in% names(N_yrs[N_yrs == i]))
Antibody_plot_data <- droplevels(Antibody_plot_data)
rm(N_yrs)
# Select variables for plot
Antibody_plot_data <- Antibody_plot_data[,c("engagement","beef_dairy","individual_antibody_result")]
# Make indivdual antibody result a factor
Antibody_plot_data$individual_antibody_result <- as.factor(Antibody_plot_data$individual_antibody_result)
levels(Antibody_plot_data$individual_antibody_result) <- list("Positive"="1", "Negative"="0")
# Make dataset long
Antibody_plot_data <- melt(Antibody_plot_data[,c("engagement","individual_antibody_result","beef_dairy")])
Antibody_plot_data$value <- 1
# Make dataset wide
Antibody_plot_data <- aggregate(. ~ engagement + individual_antibody_result + beef_dairy, data = Antibody_plot_data, FUN = sum, drop = FALSE)
# Make NA 0
Antibody_plot_data$value[is.na(Antibody_plot_data$value)] <- 0
# Make columns giving position and labels of plot labels
pos_vector <- as.vector(by(Antibody_plot_data$value, list(Antibody_plot_data$engagement, Antibody_plot_data$beef_dairy), sum))
Antibody_plot_data$pos <- c(rep(pos_vector[1:(length(pos_vector)/2)],2), rep(pos_vector[((length(pos_vector)/2)+1):length(pos_vector)],2))
Antibody_plot_data$prop <- formatC((Antibody_plot_data$value * 100) / Antibody_plot_data$pos, format="f", digits = 2) %>%
  as.numeric
# Retain only rows for positive result
Antibody_plot_data <- subset(Antibody_plot_data, individual_antibody_result=="Positive")

## Individual antigen or individual antibody testing
```

```
# Remove herds that do not conform to tissue or blood antibody testing or were mixed herd type
Antigen_antibody_plot_data <- subset(Plot_data, !is.na(test_regime2))
# Select variables for plot
Antigen_antibody_plot_data <- Antigen_antibody_plot_data[,c("engagement","beef_dairy","herd_result")]
# Make result a factor
Antigen_antibody_plot_data$herd_result <- as.factor(Antigen_antibody_plot_data$herd_result)
levels(Antigen_antibody_plot_data$herd_result) <- list("Positive"="1", "Negative"="0")
# Make dataset long
Antigen_antibody_plot_data <- melt(Antigen_antibody_plot_data[,c("engagement","herd_result","beef_dairy")])
Antigen_antibody_plot_data$value <- 1
# Make dataset wide
Antigen_antibody_plot_data <- aggregate(. ~ engagement + herd_result + beef_dairy, data = Antigen_antibody_plot_data, FUN = sum, drop = FALSE)
# Make NA 0
Antigen_antibody_plot_data$value[is.na(Antigen_antibody_plot_data$value)] <- 0
# Make columns giving position and labels of plot labels
pos_vector <- as.vector(by(Antigen_antibody_plot_data$value, list(Antigen_antibody_plot_data$engagement, Antigen_antibody_plot_data$beef_dairy), sum))
Antigen_antibody_plot_data$pos <- c(rep(pos_vector[1:(length(pos_vector)/2)],2), rep(pos_vector[((length(pos_vector)/2)+1):length(pos_vector)],2))
Antigen_antibody_plot_data$prop <- formatC((Antigen_antibody_plot_data$value * 100) / Antigen_antibody_plot_data$pos, format="f", digits = 2) %>%
  as.numeric
# Retain only rows for positive result
Antigen_antibody_plot_data <- subset(Antigen_antibody_plot_data, herd_result=="Positive")

## Combine datasets
# Make column to distinguish test type denominator
Antigen_plot_data$tests <- "Antigen"
Antibody_plot_data$tests <- "Antibody"
Antigen_antibody_plot_data$tests <- "Antigen/antibody"
# Remove result column
Antigen_plot_data$individual_antigen_result <- NULL
Antibody_plot_data$individual_antibody_result <- NULL
Antigen_antibody_plot_data$herd_result <- NULL
# Combine all test type dataframes
Plot_data <- rbind(Antigen_plot_data, Antibody_plot_data, Antigen_antibody_plot_data)

## Calculate 95% CIs
LCI <- NULL
UCI <- NULL
for(j in 1:nrow(Plot_data)){
  y <- round(prop.test(x = Plot_data[j,]$value, n = Plot_data[j,]$pos, conf.level=.95, correct=FALSE)$conf.int[1], digits = 4) * 100
  z <- round(prop.test(x = Plot_data[j,]$value, n = Plot_data[j,]$pos, conf.level=.95, correct=FALSE)$conf.int[2], digits = 4) * 100
  LCI <- c(LCI, y)
  UCI <- c(UCI, z)
}

## Edit engagement so that beef and dairy are plotted next to each other
Plot_data$engagement <- as.numeric(as.character(Plot_data$engagement))
y <- NULL
for(k in 1:nrow(Plot_data)){
  x <- ifelse(Plot_data[k,]$beef_dairy=="Beef breeder", Plot_data[k,]$engagement - 0.125, Plot_data[k,]$engagement + 0.125)
```

```r
   y <- rbind(y, x)
  }
  Plot_data$engagement <- y

  # Rename tests to make "Antigen" "Virus"
  Plot_data$tests <- as.factor(Plot_data$tests)
  levels(Plot_data$tests) <- list("Antibody" = "Antibody", "Virus" = "Antigen", "Virus/antibody" = "Antigen/antibody")

  ## Create labels for plot
  Plot_data$tests <- as.factor(Plot_data$tests)
  facet_labels <- NULL
  for(l in levels(Plot_data$tests)){
   x <- paste0(l, "\nBeef breeder ", subset(Plot_data,  beef_dairy == "Beef breeder" & tests == l)$pos[1], " Dairy ", subset(Plot_data, beef_dairy == "Dairy" & tests == l)$pos[1], sep="")
   facet_labels <- c(facet_labels, x)
  }
  names(facet_labels) <- levels(Plot_data$tests)

  # Plot
  png(filename = paste("Herd-level_test_results_for_", i, "years_of_testing_60per_virus_tested.png", sep = ""), res=600, width=5000, height=2750)
  print(ggplot(data = Plot_data, aes(x = engagement, y = prop)) +
        geom_errorbar(aes(ymax = UCI, ymin = LCI), width = 0.25) +
        geom_point(aes(color = beef_dairy, shape = beef_dairy), size = 2) +
        facet_grid(. ~ tests, labeller = labeller(tests = facet_labels)) +
        scale_colour_manual(values = c("Beef breeder" = "#CA0020", "Dairy" = "#0571B0"), name = "Herd type") +
        scale_shape_manual(values = c(19, 17), name = "Herd type") +
        scale_y_continuous(limits = c(0,60), breaks = c(0,10,20,30,40,50,60), expand = c(0, 0)) +
        scale_x_continuous(limits = c(0.5,(i + 0.5)), breaks = c(1:i), expand = c(0, 0)) +
        labs(x = "Year", y = "Percentage of herds with a positive test result") +
        theme_bw() +
        theme(legend.position = c(0.9, 0.8)))
  dev.off()
}

#### Model of herd pos/neg status - Model 1

# Select antigen testing herd years
Consec_test_data <- subset(Herd_data, !is.na(test_regime2))

# Select herds in their first set of consecutive years antigen testing
Consec_test_data  <- Consec_test_data %>%
  subset(engagement == Consec_yrs)

# Select only herds that have all their consecutive years present in the dataset
Consec_test_data <- arrange(Consec_test_data, engagement)
Consec_test_data$test_regime_engagement <- NA
for(i in 1:nrow(Consec_test_data)){
  Consec_test_data$test_regime_engagement[i] <- nrow(subset(Consec_test_data[1:i,], test_holding_number==Consec_test_data[i,]$test_holding_number))
}
Consec_test_data <- Consec_test_data %>%
```

```
  subset(test_regime_engagement == Consec_yrs)

Consec_test_data <- droplevels(Consec_test_data)

# Select herds that have at least 2 years testing
Consec_test_data <- Consec_test_data[Consec_test_data$test_holding_number %in% names(which(table(Consec_test_data$test_holding_number) != 1)), ]

### All herds
Within_herd_threshold_model <- glmer(formula = herd_result ~ Consec_yrs + (1|test_holding_number), data = Consec_test_data, family="binomial")

## Test for confounding
Confounders <- c("region", "herd_size", "beef_dairy")
Binom_mod_conf_list <- list()
Binom_mod_conf_results_table_list <- list()

for(i in Confounders){
  Binom_mod_confounders <- glmer(formula = paste0("herd_result ~ Consec_yrs + ", i, " + (1|test_holding_number)", sep=""), data = Consec_antigen_test_data, family="binomial")
  # Coefficients
  Binom_mod_confounders_results <- summary(Binom_mod_confounders)$coefficients[,1] %>%
    exp() %>%
    round(2)
  Binom_mod_confounders_Ps <- summary(Binom_mod_confounders)$coefficients[,4] %>%
    round(3)
  # 95% confidence intervals
  Binom_mod_confounders_CIs <- confint(Binom_mod_confounders, method="Wald")[-1,] %>%
    exp() %>%
    formatC(digits = 2, format = "f") %>%
    as.data.frame()
  Binom_mod_confounders_CIs$CI <- paste(Binom_mod_confounders_CIs[,1], Binom_mod_confounders_CIs[,2], sep = " - ")
  Binom_mod_confounders_results <- cbind(Binom_mod_confounders_results, Binom_mod_confounders_CIs$CI, Binom_mod_confounders_Ps)
  colnames(Binom_mod_confounders_results) <- c("OR", "CI", "P")
  # Save models
  write.csv(Binom_mod_confounders_results, paste0("Herd_pos_neg_model_results_w_", i,".csv"), row.names = TRUE)
  Binom_mod_conf_list[[i]] <- Binom_mod_confounders
  Binom_mod_conf_results_table_list[[i]] <- Binom_mod_confounders_results
}

# Model data
summary(Within_herd_threshold_model)
# Coefficients
Within_herd_threshold_model_results <- summary(Within_herd_threshold_model)$coefficients[,1] %>%
  exp() %>%
  round(2)
Within_herd_threshold_model_Ps <- summary(Within_herd_threshold_model)$coefficients[,4] %>%
  round(3)
# 95% confidence intervals
Within_herd_threshold_model_CIs <- confint(Within_herd_threshold_model, method="Wald")[-1,] %>%
  exp() %>%
  formatC(digits = 2, format = "f") %>%
```

```
  as.data.frame()
Within_herd_threshold_model_CIs$CI <- paste(Within_herd_threshold_model_CIs[,1], Within_herd_threshold_model_CIs[,2], sep = " - ")
Within_herd_threshold_model_results <- cbind(Within_herd_threshold_model_results, Within_herd_threshold_model_CIs$CI, Within_herd_threshold_model_Ps)
colnames(Within_herd_threshold_model_results) <- c("OR", "CI", "P")
# Save models
write.csv(Within_herd_threshold_model_results, "Presence_absence_of_BVD_with_continuous_testing_model.csv", row.names = TRUE)

## Test model fit

# Predict data
Within_herd_threshold_model_predict <- fitted(Within_herd_threshold_model)
# Compare predicted and observed deciles
Within_herd_threshold_model_fit_data <- data.frame(Consec_test_data$herd_result, Within_herd_threshold_model_predict)
colnames(Within_herd_threshold_model_fit_data) <- c("Observed", "Predicted")
Within_herd_threshold_model_fit_data <- Within_herd_threshold_model_fit_data %>% arrange(Predicted)
nrows_tests <- nrow(Within_herd_threshold_model_fit_data)
Within_herd_threshold_model_fit_data$Decile <- c(rep(1, round((nrows_tests / 10), digits = 0)),
                                    rep(2, (round(((2 * nrows_tests) / 10), digits = 0) - round((nrows_tests / 10), digits = 0))),
                                    rep(3, (round(((3 * nrows_tests) / 10), digits = 0) - round(((2 * nrows_tests) / 10), digits = 0))),
                                    rep(4, (round(((4 * nrows_tests) / 10), digits = 0) - round(((3 * nrows_tests) / 10), digits = 0))),
                                    rep(5, (round(((5 * nrows_tests) / 10), digits = 0) - round(((4 * nrows_tests) / 10), digits = 0))),
                                    rep(6, (round(((6 * nrows_tests) / 10), digits = 0) - round(((5 * nrows_tests) / 10), digits = 0))),
                                    rep(7, (round(((7 * nrows_tests) / 10), digits = 0) - round(((6 * nrows_tests) / 10), digits = 0))),
                                    rep(8, (round(((8 * nrows_tests) / 10), digits = 0) - round(((7 * nrows_tests) / 10), digits = 0))),
                                    rep(9, (round(((9 * nrows_tests) / 10), digits = 0) - round(((8 * nrows_tests) / 10), digits = 0))),
                                    rep(10, (nrows_tests - round(((9 * nrows_tests) / 10), digits = 0)))) %>%
  as.factor()
Within_herd_threshold_model_fit_data <- melt(Within_herd_threshold_model_fit_data)
Within_herd_threshold_model_fit_decile_data <- aggregate(value ~ Decile + variable, data = Within_herd_threshold_model_fit_data, FUN = mean)
Within_herd_threshold_model_fit_herd_size_data <- aggregate(value ~ Herd_size + variable, data = Within_herd_threshold_model_fit_data, FUN = mean)
Within_herd_threshold_model_fit_region_data <- aggregate(value ~ Region + variable, data = Within_herd_threshold_model_fit_data, FUN = mean)

# Round proportion
Within_herd_threshold_model_fit_decile_data$value <- round(Within_herd_threshold_model_fit_decile_data$value, 2)

# Plot
png(filename="Model_1_herd-level_prev_fit.png", res=600, width=5000, height=2750)
print(ggplot(Within_herd_threshold_model_fit_decile_data, aes(x = Decile, y = value, fill = variable), xlab = "Decile") +
      geom_bar(stat = "identity", position = "dodge") +
      scale_fill_manual(values = c("Observed"="#E69F00", "Predicted"="#56B4E9"), name = "Data") +
      geom_text(aes(label = formatC(value, format="f", digits = 2)), position = position_dodge(width = 0.9), vjust = -0.25) +
      theme_bw())
dev.off()

#### Alluvial plot of herd status across 5 yrs of testing - Figure 4

## Number of consecutive years
Plot_data <- subset(Consec_test_data, Consec_yrs < 6)
subset_levels <- Plot_data$test_holding_number %>%
```

```
  table() %>%
  as.data.frame() %>%
  subset(Freq==5) %>%
  pull(1)
Plot_data <- subset(Plot_data, test_holding_number %in% subset_levels)
Plot_data <- Plot_data[,c("test_holding_number", "herd_result", "Consec_yrs")]
Plot_data$Consec_yrs <- as.factor(Plot_data$Consec_yrs)
Plot_data <- Plot_data %>%
  pivot_wider(names_from = Consec_yrs, values_from = herd_result)
colnames(Plot_data) <- c("test_holding_number", "first", "second", "third", "fourth", "fifth")
Plot_data <- lapply(Plot_data, as.factor) %>% as.data.frame()
Plot_data <- plyr::count(Plot_data[,-1])
for(i in 1:5){
  levels(Plot_data[,i]) <- list("Pos." = "1", "Neg." = "0")
}
Plot_data$colour <- NA
Plot_data$colour[Plot_data$first=="Pos." & Plot_data$fifth=="Pos."] <- "1"
Plot_data$colour[Plot_data$first=="Pos." & Plot_data$fifth=="Neg."] <- "2"
Plot_data$colour[Plot_data$first=="Neg." & Plot_data$fifth=="Pos."] <- "3"
Plot_data$colour[Plot_data$first=="Neg." & Plot_data$fifth=="Neg."] <- "4"

write.csv(Plot_data, "Alluvial_plot_data_5_consec_yrs_testing.csv")

## Calculate proportion of herds going consistently positive, consistently negative or fluctuating
# Pos - Pos
sum(subset(Plot_data, first=="Pos."&fifth=="Pos.")$freq)
(sum(subset(Plot_data, first=="Pos."&fifth=="Pos.")$freq))*100 / sum(subset(Plot_data, first=="Pos.")$freq)
# Pos - Neg
sum(subset(Plot_data, first=="Pos."&fifth=="Neg.")$freq)
(sum(subset(Plot_data, first=="Pos."&fifth=="Neg.")$freq))*100 / sum(subset(Plot_data, first=="Pos.")$freq)
# Neg - Pos
sum(subset(Plot_data, first=="Neg."&fifth=="Pos.")$freq)
(sum(subset(Plot_data, first=="Neg."&fifth=="Pos.")$freq))*100 / sum(subset(Plot_data, first=="Neg.")$freq)
# Neg - Neg
sum(subset(Plot_data, first=="Neg."&fifth=="Neg.")$freq)
(sum(subset(Plot_data, first=="Neg."&fifth=="Neg.")$freq))*100 / sum(subset(Plot_data, first=="Neg.")$freq)

# Change factor levels to include %
for(i in colnames(Plot_data)[1:5]){
  levels(Plot_data[,i]) <- c(paste0("Pos.\n", round((sum(subset(Plot_data, pull(Plot_data, i)=="Pos.")$freq)*100)/sum(Plot_data$freq), digits=1), "%", sep=""),
                             paste0("Neg.\n", round((sum(subset(Plot_data, pull(Plot_data, i)=="Neg.")$freq)*100)/sum(Plot_data$freq), digits=1), "%", sep=""))
}

png(filename="Alluvial_plot_consecutive_yrs.png", res=600, width=5000, height=4000)
ggplot(data = Plot_data,
       aes(axis1 = first, axis2 = second, axis3 = third, axis4 = fourth, axis5 = fifth, y = freq)) +
  geom_alluvium(aes(fill=colour)) +
  geom_stratum() +
  geom_text(stat = "stratum",
```

```
      aes(label = after_stat(stratum))) +
  scale_x_discrete(limits = c("First year", "Second year", "Third year", "Fourth year", "Fifth year"),
          expand = c(0.15, 0.05)) +
  labs(y = "Frequency") +
  scale_fill_viridis_d() +
  theme_bw() +
  theme(legend.position="none", axis.text = element_text(size = 12), axis.title = element_text(size = 12))
dev.off()

#### Individual test results ####

## Proportion of antigen positive tests for antigen testing herds - Supplementary Figure 3

# Select only individual antigen tests (tissue/blood) - all antigen testing herds
Plot_data <- subset(Data, (sample_type=="Tissue" | sample_type=="Blood") & analysis_type=="BVDAg/v" & pooled_sample=="0")
Plot_data2 <- subset(Herd_data, test_regime2=="Antigen")
Plot_data2 <- Plot_data2[,c("test_holding_number","sample_year")]
Plot_data <- inner_join(Plot_data, Plot_data2, by=c("test_holding_number","sample_year"))
Plot_data <- Plot_data[,c("sample_year","antigen_result")]
# Remove tests with no result
Plot_data <- subset(Plot_data, !is.na(antigen_result))
# Make result factor and label
Plot_data$antigen_result <- as.factor(Plot_data$antigen_result)
levels(Plot_data$antigen_result) <- list("Positive" = "1", "Negative" = "0")
# Make a count column
Plot_data$value <- 1
# Make dataset wide
Plot_data <- aggregate(. ~ sample_year + antigen_result, data = Plot_data, FUN = sum)
# Make columns giving position and labels of plot labels
pos_vector <- as.vector(by(Plot_data$value, Plot_data$sample_year, sum))
# Retain only rows for positive result
Plot_data <- subset(Plot_data, antigen_result=="Positive")
Plot_data$pos <- pos_vector
Plot_data$prop <- formatC((Plot_data$value * 100) / Plot_data$pos, format="f", digits = 2)

LCI <- NULL
UCI <- NULL
for(j in 1:nrow(Plot_data)){
  y <- round(prop.test(x = Plot_data[j,]$value, n = Plot_data[j,]$pos, conf.level=.95, correct=FALSE)$conf.int[1], digits = 4) * 100
  z <- round(prop.test(x = Plot_data[j,]$value, n = Plot_data[j,]$pos, conf.level=.95, correct=FALSE)$conf.int[2], digits = 4) * 100
  LCI <- c(LCI, y)
  UCI <- c(UCI, z)
}

Plot_data <- cbind(Plot_data, LCI, UCI)

## Proportion of antigen positive tests in positive herds

# Select only individual antigen tests (tissue/blood) - all antigen testing herds
```

```
Plot_data2 <- subset(Data, (sample_type=="Tissue" | sample_type=="Blood") & analysis_type=="BVDAg/v" & pooled_sample=="0")
Plot_data22 <- subset(Herd_data, test_regime2=="Antigen" & herd_result==1)
Plot_data22 <- Plot_data22[,c("test_holding_number","sample_year")]
Plot_data2 <- inner_join(Plot_data2, Plot_data22, by=c("test_holding_number","sample_year"))
Plot_data2 <- Plot_data2[,c("sample_year","antigen_result")]
# Remove tests with no result
Plot_data2 <- subset(Plot_data2, !is.na(antigen_result))
# Make result factor and label
Plot_data2$antigen_result <- as.factor(Plot_data2$antigen_result)
levels(Plot_data2$antigen_result) <- list("Positive" = "1", "Negative" = "0")
# Make a count column
Plot_data2$value <- 1
# Make dataset wide
Plot_data2 <- aggregate(. ~ sample_year + antigen_result, data = Plot_data2, FUN = sum)
# Make columns giving position and labels of plot labels
pos_vector <- as.vector(by(Plot_data2$value, Plot_data2$sample_year, sum))
# Retain only reows for positive result
Plot_data2 <- subset(Plot_data2, antigen_result=="Positive")
Plot_data2$pos <- pos_vector
Plot_data2$prop <- formatC((Plot_data2$value * 100) / Plot_data2$pos, format="f", digits = 2)

LCI <- NULL
UCI <- NULL
for(j in 1:nrow(Plot_data2)){
  y <- round(prop.test(x = Plot_data2[j,]$value, n = Plot_data2[j,]$pos, conf.level=.95, correct=FALSE)$conf.int[1], digits = 4) * 100
  z <- round(prop.test(x = Plot_data2[j,]$value, n = Plot_data2[j,]$pos, conf.level=.95, correct=FALSE)$conf.int[2], digits = 4) * 100
  LCI <- c(LCI, y)
  UCI <- c(UCI, z)
}

Plot_data2 <- cbind(Plot_data2, LCI, UCI)

# Merge datasets
Plot_data$results <- "All"
Plot_data2$results <- "Positives"
Plot_data <- rbind(Plot_data, Plot_data2)
Plot_data$antigen_result <- NULL
Plot_data$prop <- as.numeric(Plot_data$prop)
# Rename denominator groups
Plot_data$results <- as.factor(Plot_data$results)
levels(Plot_data$results) <- list("Virus testing herds" = "All", "Virus positive herds" = "Positives")
Plot_data$results <- relevel(Plot_data$results, ref = "Virus positive herds")
write.csv(Plot_data, "Prop_of_virus_tests_pos.csv")

# Plot
png(filename="Test-level_antigen_test_results.png", res=600, width=4000, height=3000)
ggplot(data = Plot_data, aes(x = as.numeric(as.character(sample_year)), y = prop)) +
  geom_errorbar(aes(ymax = UCI, ymin = LCI), width = 0.25) +
  geom_point(aes(color = results, shape = results), size = 2) +
```

```r
  scale_colour_manual(values = c("Virus positive herds"="#68228B", "Virus testing herds"="#008B00"), name = "Herds") +
  scale_shape_manual(values = c(19, 17), name = "Herds") +
  scale_x_continuous(breaks = c(seq(2016, as.numeric(tail(levels(Plot_data$sample_year), n=1)), 1))) +
  scale_y_continuous(limits = c(0,2.75), breaks = c(0,0.5,1,1.5,2,2.5), expand = c(0,0)) +
  labs(x = "Year", y = "Percentage of virus tests with a positive result") +
  theme_bw()
dev.off()

## Proportion of antigen positive tests for antigen testing herds - Supplementary Figure 3

# Select only individual antigen tests (tissue/blood) - all antigen testing herds
Plot_data <- subset(Data, (sample_type=="Tissue" | sample_type=="Blood") & analysis_type=="BVDAg/v" & pooled_sample=="0")
Plot_data2 <- subset(Herd_data, test_regime2=="Antigen")
Plot_data2 <- Plot_data2[,c("test_holding_number","sample_year")]
Plot_data <- inner_join(Plot_data, Plot_data2, by=c("test_holding_number","sample_year"))
Plot_data <- Plot_data[,c("sample_year","antigen_result")]
# Remove tests with no result
Plot_data <- subset(Plot_data, !is.na(antigen_result))
# Make result factor and label
Plot_data$antigen_result <- as.factor(Plot_data$antigen_result)
levels(Plot_data$antigen_result) <- list("Positive" = "1", "Negative" = "0")
# Make a count column
Plot_data$value <- 1
# Make dataset wide
Plot_data <- aggregate(. ~ sample_year + antigen_result, data = Plot_data, FUN = sum)
# Make columns giving position and labels of plot labels
pos_vector <- as.vector(by(Plot_data$value, Plot_data$sample_year, sum))
# Retain only rows for positive result
Plot_data <- subset(Plot_data, antigen_result=="Positive")
Plot_data$pos <- pos_vector
Plot_data$prop <- formatC((Plot_data$value * 100) / Plot_data$pos, format="f", digits = 2)

LCI <- NULL
UCI <- NULL
for(j in 1:nrow(Plot_data)){
  y <- round(prop.test(x = Plot_data[j,]$value, n = Plot_data[j,]$pos, conf.level=.95, correct=FALSE)$conf.int[1], digits = 4) * 100
  z <- round(prop.test(x = Plot_data[j,]$value, n = Plot_data[j,]$pos, conf.level=.95, correct=FALSE)$conf.int[2], digits = 4) * 100
  LCI <- c(LCI, y)
  UCI <- c(UCI, z)
}

Plot_data <- cbind(Plot_data, LCI, UCI)

## Proportion of antigen positive tests in positive herds - no min antigen tests

# Select only individual antigen tests (tissue/blood) - all antigen testing herds
Plot_data2 <- subset(Data, (sample_type=="Tissue" | sample_type=="Blood") & analysis_type=="BVDAg/v" & pooled_sample=="0")
Plot_data22 <- subset(Herd_data, test_regime=="Antigen" & herd_result==1)
Plot_data22 <- Plot_data22[,c("test_holding_number","sample_year")]
```

```
Plot_data2 <- inner_join(Plot_data2, Plot_data22, by=c("test_holding_number","sample_year"))
Plot_data2 <- Plot_data2[,c("sample_year","antigen_result")]
# Remove tests with no result
Plot_data2 <- subset(Plot_data2, !is.na(antigen_result))
# Make result factor and label
Plot_data2$antigen_result <- as.factor(Plot_data2$antigen_result)
levels(Plot_data2$antigen_result) <- list("Positive" = "1", "Negative" = "0")
# Make a count column
Plot_data2$value <- 1
# Make dataset wide
Plot_data2 <- aggregate(. ~ sample_year + antigen_result, data = Plot_data2, FUN = sum)
# Make columns giving position and labels of plot labels
pos_vector <- as.vector(by(Plot_data2$value, Plot_data2$sample_year, sum))
# Retain only reows for positive result
Plot_data2 <- subset(Plot_data2, antigen_result=="Positive")
Plot_data2$pos <- pos_vector
Plot_data2$prop <- formatC((Plot_data2$value * 100) / Plot_data2$pos, format="f", digits = 2)

LCI <- NULL
UCI <- NULL
for(j in 1:nrow(Plot_data2)){
  y <- round(prop.test(x = Plot_data2[j,]$value, n = Plot_data2[j,]$pos, conf.level=.95, correct=FALSE)$conf.int[1], digits = 4) * 100
  z <- round(prop.test(x = Plot_data2[j,]$value, n = Plot_data2[j,]$pos, conf.level=.95, correct=FALSE)$conf.int[2], digits = 4) * 100
  LCI <- c(LCI, y)
  UCI <- c(UCI, z)
}

Plot_data2 <- cbind(Plot_data2, LCI, UCI)

# Merge datasets
Plot_data$results <- "All"
Plot_data2$results <- "Positives"
Plot_data <- rbind(Plot_data, Plot_data2)
Plot_data$antigen_result <- NULL
Plot_data$prop <- as.numeric(Plot_data$prop)
# Rename denominator groups
Plot_data$results <- as.factor(Plot_data$results)
levels(Plot_data$results) <- list("Virus testing herds" = "All", "Virus positive herds" = "Positives")
Plot_data$results <- relevel(Plot_data$results, ref = "Virus positive herds")
write.csv(Plot_data, "Prop_of_virus_tests_pos.csv")

# Plot
png(filename="Test-level_antigen_test_results_no_min_tests.png", res=600, width=4000, height=3000)
ggplot(data = Plot_data, aes(x = as.numeric(as.character(sample_year)), y = prop)) +
  geom_errorbar(aes(ymax = UCI, ymin = LCI), width = 0.25) +
  geom_point(aes(color = results, shape = results), size = 2) +
  scale_colour_manual(values = c("Virus positive herds"="#68228B", "Virus testing herds"="#008B00"), name = "Herds") +
  scale_shape_manual(values = c(19, 17), name = "Herds") +
  scale_x_continuous(breaks = c(seq(2016, as.numeric(tail(levels(Plot_data$sample_year), n=1)), 1))) +
```

```
  scale_y_continuous(limits = c(0,2.75), breaks = c(0,0.5,1,1.5,2,2.5), expand = c(0,0)) +
  labs(x = "Year", y = "Percentage of virus tests with a positive result") +
  theme_bw()
dev.off()

#### Proportion of antigen positive tests with consecutive years testing - Figure 5

## Select only antigen testing herds
Antigen_testing_herds_data <- subset(Herd_data, test_regime2=="Antigen")
## Select only first testing bout for each herd
Antigen_testing_herds_data <- Antigen_testing_herds_data %>%
  subset(Consec_yrs==engagement)

# Dataframe of antigen tests
Ind_antigen_tests <- subset(Data, (sample_type=="Tissue" | sample_type=="Blood") & analysis_type=="BVDAg/v" & pooled_sample=="0")

## Continue with herds with different numbers of years testing
for(i in 3:5){
  Antigen_testing_herds <- Antigen_testing_herds_data %>%
    subset(engagement <= i) %>%
    droplevels()
  Antigen_testing_herds$engagement <- as.factor(Antigen_testing_herds$engagement)
  # Remove herds that did not consecutively antigen test
  N_yrs <- table(Antigen_testing_herds$test_holding_number)
  Antigen_testing_herds <- Antigen_testing_herds %>%
    subset(test_holding_number %in% names(N_yrs[N_yrs == i]))
  Antigen_testing_herds <- droplevels(Antigen_testing_herds)
  rm(N_yrs)

  # Create dataframe of positive herd-years
  Antigen_testing_herds_pos_yr1_herds <- Antigen_testing_herds %>%
    subset(herd_result=="1" & Consec_yrs=="1") %>%
    droplevels()
  Antigen_testing_herds_pos_yr1 <- subset(Antigen_testing_herds, test_holding_number %in% levels(Antigen_testing_herds_pos_yr1_herds$test_holding_number))
  Antigen_testing_herds <- Antigen_testing_herds[,c("test_holding_number", "sample_year", "Consec_yrs")]
  Antigen_testing_herds_pos_yr1 <- Antigen_testing_herds_pos_yr1[,c("test_holding_number", "sample_year", "Consec_yrs")]

  #### Proportion of antigen positive tests for antigen testing herds
  Plot_data <- inner_join(Ind_antigen_tests, Antigen_testing_herds, by=c("test_holding_number","sample_year")) %>%
    droplevels()
  Plot_data <- Plot_data[,c("Consec_yrs","antigen_result")]
  # Remove tests with no result
  Plot_data <- subset(Plot_data, !is.na(antigen_result))
  # Make result factor and label
  Plot_data$antigen_result <- as.factor(Plot_data$antigen_result)
  levels(Plot_data$antigen_result) <- list("Positive" = "1", "Negative" = "0")
  # Make a count column
  Plot_data$value <- 1
  # Make dataset wide
```

```
Plot_data <- aggregate(. ~ Consec_yrs + antigen_result, data = Plot_data, FUN = sum)
# Make columns giving position and labels of plot labels
pos_vector <- as.vector(by(Plot_data$value, Plot_data$Consec_yrs, sum))
# Retain only rows for positive result
Plot_data <- subset(Plot_data, antigen_result=="Positive")
Plot_data$pos <- pos_vector
Plot_data$prop <- formatC((Plot_data$value * 100) / Plot_data$pos, format="f", digits = 2)

LCI <- NULL
UCI <- NULL
for(j in 1:nrow(Plot_data)){
  y <- round(prop.test(x = Plot_data[j,]$value, n = Plot_data[j,]$pos, conf.level=.95, correct=FALSE)$conf.int[1], digits = 4) * 100
  z <- round(prop.test(x = Plot_data[j,]$value, n = Plot_data[j,]$pos, conf.level=.95, correct=FALSE)$conf.int[2], digits = 4) * 100
  LCI <- c(LCI, y)
  UCI <- c(UCI, z)
}

Plot_data <- cbind(Plot_data, LCI, UCI)

## Proportion of antigen positive tests in positive herds

# Select only individual antigen tests (tissue/blood) - all antigen testing herds
Plot_data2 <- inner_join(Ind_antigen_tests, Antigen_testing_herds_pos_yr1, by=c("test_holding_number","sample_year")) %>%
  droplevels()
Plot_data2 <- Plot_data2[,c("Consec_yrs","antigen_result")]

# Remove tests with no result
Plot_data2 <- subset(Plot_data2, !is.na(antigen_result))
# Make result factor and label
Plot_data2$antigen_result <- as.factor(Plot_data2$antigen_result)
levels(Plot_data2$antigen_result) <- list("Positive" = "1", "Negative" = "0")
# Make a count column
Plot_data2$value <- 1
# Make dataset wide
Plot_data2 <- aggregate(. ~ Consec_yrs + antigen_result, data = Plot_data2, FUN = sum)
# Make columns giving position and labels of plot labels
pos_vector <- as.vector(by(Plot_data2$value, Plot_data2$Consec_yrs, sum))
# Retain only reows for positive result
Plot_data2 <- subset(Plot_data2, antigen_result=="Positive")
Plot_data2$pos <- pos_vector
Plot_data2$prop <- formatC((Plot_data2$value * 100) / Plot_data2$pos, format="f", digits = 4)

LCI <- NULL
UCI <- NULL
for(j in 1:nrow(Plot_data2)){
  y <- round(prop.test(x = Plot_data2[j,]$value, n = Plot_data2[j,]$pos, conf.level=.95, correct=FALSE)$conf.int[1], digits = 4) * 100
  z <- round(prop.test(x = Plot_data2[j,]$value, n = Plot_data2[j,]$pos, conf.level=.95, correct=FALSE)$conf.int[2], digits = 4) * 100
  LCI <- c(LCI, y)
  UCI <- c(UCI, z)
```

```
  }

  Plot_data2 <- cbind(Plot_data2, LCI, UCI)

  # Merge datasets
  Plot_data$results <- "All"
  Plot_data2$results <- "Positives"
  Plot_data <- rbind(Plot_data, Plot_data2)
  Plot_data$antigen_result <- NULL
  Plot_data$prop <- as.numeric(Plot_data$prop)
  # Rename denominator groups
  Plot_data$results <- as.factor(Plot_data$results)
  levels(Plot_data$results) <- list("Virus testing herds" = "All", "Virus positive herds" = "Positives")
  Plot_data$results <- relevel(Plot_data$results, ref = "Virus positive herds")
  write.csv(Plot_data, paste0("Prop_of_virus_tests_pos_", i, "_consec_yrs_testing.csv", sep=""))

  Plot_data$Consec_yrs <- as.numeric(Plot_data$Consec_yrs)
  y <- NULL
  for(j in 1:nrow(Plot_data)){
   x <- ifelse(Plot_data[j,]$results=="Virus testing herds", Plot_data[j,]$Consec_yrs - 0.125, Plot_data[j,]$Consec_yrs + 0.125)
   y <- rbind(y, x)
  }
  Plot_data$Consec_yrs <- y

  # Plot
  png(filename=paste0("Test-level_antigen_test_results_", i, "_consec_yrs_testing.png", sep=""), res=600, width=4000, height=2500)
  print(ggplot(data = Plot_data, aes(x = as.numeric(as.character(Consec_yrs)), y = prop)) +
          geom_errorbar(aes(ymax = UCI, ymin = LCI), width = 0.25) +
          geom_point(aes(color = results, shape = results), size = 2) +
          scale_colour_manual(values = c("Virus positive herds"="#68228B", "Virus testing herds"="#008B00"), name = "Herds") +
          scale_shape_manual(values = c(19, 17), name = "Herds") +
          scale_x_continuous(breaks = c(seq(1, as.numeric(tail(levels(as.factor(Plot_data$Consec_yrs)), n=1)), 1))) +
          scale_y_continuous(limits = c(0,2.5), breaks = c(0,0.5,1,1.5,2), expand = c(0,0)) +
          labs(x = "Year", y = "Percentage of virus tests with a positive result") +
          theme_bw())
  dev.off()
}

#### Decrease in test positive within-herd prevalence over time - Model 2

# Select antigen testing herd years
Consec_antigen_test_data <- subset(Herd_data, test_regime2=="Antigen")

# Select herds in their first set of consecutive years antigen testing
Consec_antigen_test_data  <- Consec_antigen_test_data %>%
  subset(engagement == Consec_yrs)

# Select only herds that have all their consecutive years present in the dataset
Consec_antigen_test_data <- arrange(Consec_antigen_test_data, engagement)
```

```
Consec_antigen_test_data$antigen_engagement <- NA
for(i in 1:nrow(Consec_antigen_test_data)){
  Consec_antigen_test_data$antigen_engagement[i] <- nrow(subset(Consec_antigen_test_data[1:i,], test_holding_number==Consec_antigen_test_data[i,]$test_holding_number))
}
Consec_antigen_test_data  <- Consec_antigen_test_data %>%
  subset(antigen_engagement == Consec_yrs)

Consec_antigen_test_data <- droplevels(Consec_antigen_test_data)

# Select herds that have at least 2 years testing
Consec_antigen_test_data <- Consec_antigen_test_data[Consec_antigen_test_data$test_holding_number %in% names(which(table(Consec_antigen_test_data$test_holding_number) != 1)), ]

### Full model
Within_herd_prop_model <- glmer(formula = prop_antigen_tests_pos ~ Consec_yrs + (1|test_holding_number), weights = individual_antigen, data = Consec_antigen_test_data, family="binomial")

# Convergence warnings - see if these are a problem

## 1. decrease stopping tolerances: NA

## 2. center and scale predictors: NA

## 3. recompute gradient and Hessian with Richardson extrapolation: Similar result
devfun <- update(Within_herd_prop_model, devFunOnly=TRUE)
if (isLMM(Within_herd_prop_model)) {
  pars <- getME(Within_herd_prop_model,"theta")
} else {
  ## GLMM: requires both random and fixed parameters
  pars <- getME(Within_herd_prop_model, c("theta","fixef"))
}
if (require("numDeriv")) {
  cat("hess:\n"); print(hess <- hessian(devfun, unlist(pars)))
  cat("grad:\n"); print(grad <- grad(devfun, unlist(pars)))
  cat("scaled gradient:\n")
  print(scgrad <- solve(chol(hess), grad))
}
## compare with internal calculations: No change
Within_herd_prop_model@optinfo$derivs

## compute reciprocal condition number of Hessian
H <- fm1@optinfo$derivs$Hessian
Matrix::rcond(H)

## 4. restart the fit from the original value (or a slightly perturbed value): NA - not numeric

## 5. try all available optimizers
Within_herd_prop_model.all <- allFit(Within_herd_prop_model)
ss <- summary(Within_herd_prop_model.all)
ss$ fixef              ## fixed effects
ss$ llik               ## log-likelihoods
```

```
ss$ sdcor              ## SDs and correlations
ss$ theta              ## Cholesky factors
ss$ which.OK           ## which fits worked

## Outputs are similar in all optimisers, therefore confident that the model has converged

## Test for confounding
Confounders <- c("region", "herd_size", "beef_dairy")
Prop_binom_mod_conf_list <- list()
Prop_binom_mod_conf_results_table_list <- list()

for(i in Confounders){
  Prop_binom_mod_confounders <- glmer(formula = paste0("prop_antigen_tests_pos ~ Consec_yrs + ", i, " + (1|test_holding_number)", sep=""), weights = individual_antigen, data = Consec_antigen_test_data, family="binomial")
  # Coefficients
  Prop_binom_mod_confounders_results <- summary(Prop_binom_mod_confounders)$coefficients[,1] %>%
    exp() %>%
    round(2)
  Prop_binom_mod_confounders_Ps <- summary(Prop_binom_mod_confounders)$coefficients[,4] %>%
    round(3)
  # 95% confidence intervals
  Prop_binom_mod_confounders_CIs <- confint(Prop_binom_mod_confounders, method="Wald")[-1,] %>%
    exp() %>%
    formatC(digits = 2, format = "f") %>%
    as.data.frame()
  Prop_binom_mod_confounders_CIs$CI <- paste(Prop_binom_mod_confounders_CIs[,1], Prop_binom_mod_confounders_CIs[,2], sep = " - ")
  Prop_binom_mod_confounders_results <- cbind(Prop_binom_mod_confounders_results, Prop_binom_mod_confounders_CIs$CI, Prop_binom_mod_confounders_Ps)
  colnames(Prop_binom_mod_confounders_results) <- c("OR", "CI", "P")
  # Save models
  write.csv(Prop_binom_mod_confounders_results, paste0("First_yr_pos_or_neg_within_herd_pos_change_model_results_w_", i,".csv"), row.names = TRUE)
  Prop_binom_mod_conf_list[[i]] <- Prop_binom_mod_confounders
  Prop_binom_mod_conf_results_table_list[[i]] <- Prop_binom_mod_confounders_results
}

summary(Within_herd_prop_model)
# Coefficients
Within_herd_prop_model_results <- summary(Within_herd_prop_model)$coefficients[,1] %>%
  exp() %>%
  round(2)
Within_herd_prop_model_Ps <- summary(Within_herd_prop_model)$coefficients[,4] %>%
  round(3)
# 95% confidence intervals
Within_herd_prop_model_CIs <- confint(Within_herd_prop_model, method="Wald")[-1,] %>%
  exp() %>%
  formatC(digits = 2, format = "f") %>%
  as.data.frame()
Within_herd_prop_model_CIs$CI <- paste(Within_herd_prop_model_CIs[,1], Within_herd_prop_model_CIs[,2], sep = " - ")
Within_herd_prop_model_results <- cbind(Within_herd_prop_model_results, Within_herd_prop_model_CIs$CI, Within_herd_prop_model_Ps)
colnames(Within_herd_prop_model_results) <- c("OR", "CI", "P")
# Save models
```

```
write.csv(Within_herd_prop_model_results, "Within_herd_prevalence_of_BVD_model.csv", row.names = TRUE)

## Test model fit

# Predict data
Within_herd_prop_model_predict <- fitted(Within_herd_prop_model)
# Compare predicted and observed deciles
Within_herd_prop_model_fit_data <- data.frame(Consec_antigen_test_data$prop_antigen_tests_pos, Within_herd_prop_model_predict)
colnames(Within_herd_prop_model_fit_data) <- c("Observed", "Predicted")
Within_herd_prop_model_fit_data <- Within_herd_prop_model_fit_data %>% arrange(Predicted)
nrows_tests <- nrow(Within_herd_prop_model_fit_data)
Within_herd_prop_model_fit_data$Decile <- c(rep(1, round((nrows_tests / 10), digits = 0)),
                                      rep(2, (round(((2 * nrows_tests) / 10), digits = 0) - round((nrows_tests / 10), digits = 0))),
                                      rep(3, (round(((3 * nrows_tests) / 10), digits = 0) - round(((2 * nrows_tests) / 10), digits = 0))),
                                      rep(4, (round(((4 * nrows_tests) / 10), digits = 0) - round(((3 * nrows_tests) / 10), digits = 0))),
                                      rep(5, (round(((5 * nrows_tests) / 10), digits = 0) - round(((4 * nrows_tests) / 10), digits = 0))),
                                      rep(6, (round(((6 * nrows_tests) / 10), digits = 0) - round(((5 * nrows_tests) / 10), digits = 0))),
                                      rep(7, (round(((7 * nrows_tests) / 10), digits = 0) - round(((6 * nrows_tests) / 10), digits = 0))),
                                      rep(8, (round(((8 * nrows_tests) / 10), digits = 0) - round(((7 * nrows_tests) / 10), digits = 0))),
                                      rep(9, (round(((9 * nrows_tests) / 10), digits = 0) - round(((8 * nrows_tests) / 10), digits = 0))),
                                      rep(10, (nrows_tests - round(((9 * nrows_tests) / 10), digits = 0)))) %>%
  as.factor()
Within_herd_prop_model_fit_data <- melt(Within_herd_prop_model_fit_data)
Within_herd_prop_model_fit_decile_data <- aggregate(value ~ Decile + variable, data = Within_herd_prop_model_fit_data, FUN = mean)
Within_herd_prop_model_fit_herd_size_data <- aggregate(value ~ Herd_size + variable, data = Within_herd_prop_model_fit_data, FUN = mean)
Within_herd_prop_model_fit_region_data <- aggregate(value ~ Region + variable, data = Within_herd_prop_model_fit_data, FUN = mean)

# Round proportion
Within_herd_prop_model_fit_decile_data$value <- round(Within_herd_prop_model_fit_decile_data$value, 2)

# Plot
png(filename="Model_2_within_herd_prev_fit.png", res=600, width=5000, height=2750)
print(ggplot(Within_herd_prop_model_fit_decile_data, aes(x = Decile, y = value, fill = variable), xlab = "Decile") +
    geom_bar(stat = "identity", position = "dodge") +
    scale_fill_manual(values = c("Observed"="#E69F00", "Predicted"="#56B4E9"), name = "Data") +
    geom_text(aes(label = formatC(value, format="f", digits = 2)), position = position_dodge(width = 0.9), vjust = -0.25) +
    theme_bw())
dev.off()
```
